# Variation in stem mortality rates determines patterns of above‐ground biomass in Amazonian forests: implications for dynamic global vegetation models

**DOI:** 10.1111/gcb.13315

**Published:** 2016-05-19

**Authors:** Michelle O. Johnson, David Galbraith, Manuel Gloor, Hannes De Deurwaerder, Matthieu Guimberteau, Anja Rammig, Kirsten Thonicke, Hans Verbeeck, Celso von Randow, Abel Monteagudo, Oliver L. Phillips, Roel J. W. Brienen, Ted R. Feldpausch, Gabriela Lopez Gonzalez, Sophie Fauset, Carlos A. Quesada, Bradley Christoffersen, Philippe Ciais, Gilvan Sampaio, Bart Kruijt, Patrick Meir, Paul Moorcroft, Ke Zhang, Esteban Alvarez‐Davila, Atila Alves de Oliveira, Ieda Amaral, Ana Andrade, Luiz E. O. C. Aragao, Alejandro Araujo‐Murakami, Eric J. M. M. Arets, Luzmila Arroyo, Gerardo A. Aymard, Christopher Baraloto, Jocely Barroso, Damien Bonal, Rene Boot, Jose Camargo, Jerome Chave, Alvaro Cogollo, Fernando Cornejo Valverde, Antonio C. Lola da Costa, Anthony Di Fiore, Leandro Ferreira, Niro Higuchi, Euridice N. Honorio, Tim J. Killeen, Susan G. Laurance, William F. Laurance, Juan Licona, Thomas Lovejoy, Yadvinder Malhi, Bia Marimon, Ben Hur Marimon, Darley C. L. Matos, Casimiro Mendoza, David A. Neill, Guido Pardo, Marielos Peña‐Claros, Nigel C. A. Pitman, Lourens Poorter, Adriana Prieto, Hirma Ramirez‐Angulo, Anand Roopsind, Agustin Rudas, Rafael P. Salomao, Marcos Silveira, Juliana Stropp, Hans ter Steege, John Terborgh, Raquel Thomas, Marisol Toledo, Armando Torres‐Lezama, Geertje M. F. van der Heijden, Rodolfo Vasquez, Ima Cèlia Guimarães Vieira, Emilio Vilanova, Vincent A. Vos, Timothy R. Baker

**Affiliations:** ^1^ School of Geography University of Leeds Leeds LS6 2QT UK; ^2^ CAVElab Computational & Applied Vegetation Ecology Faculty of Bioscience Engineering Ghent University Coupure Links 653 B‐9000 Gent Belgium; ^3^ Laboratoire des Sciences du Climat et de l'Environnement, LSCE/IPSL, CEA‐CNRS‐UVSQ Université Paris‐Saclay F‐91191 Gif‐sur‐Yvette France; ^4^ UMR 7619 METIS IPSL, Sorbonne Universités, UPMC, CNRS, EPHE 75252 Paris France; ^5^ TUM School of Life Sciences Weihenstephan Technical University Munich Hans‐Carl‐von‐Carlowitz‐Platz 2 85354 Freising Germany; ^6^ Potsdam Institute for Climate Impact Research (PIK) Telegrafenberg A62 PO Box 60 12 03 D‐14412 Potsdam Germany; ^7^ INPE Av. Dos Astronautas, 1.758, Jd. Granja CEP: 12227‐010 Sao Jose dos Campos SP Brazil; ^8^ Jardín Botánico de Missouri Prolongacion Bolognesi Mz.e, Lote 6 Oxapampa, Pasco Peru; ^9^ Geography College of Life and Environmental Sciences University of Exeter Rennes Drive Exeter EX4 4RJ UK; ^10^ INPA Av. André Araújo, 2.936 CEP 69067‐375 Petrópolis, Manaus AM Brazil; ^11^ School of Geosciences University of Edinburgh Edinburgh EH9 3FF UK; ^12^ Earth and Environmental Sciences Division Los Alamos National Laboratory PO Box 1663 Los Alamos NM 87545 USA; ^13^ ALTERRA Wageningen‐UR PO Box 47 6700 AA Wageningen The Netherlands; ^14^ Research School of Biology Australian National University Canberra ACT 0200 Australia; ^15^ Department of Organismic and Evolutionary Biology Harvard University 26 Oxford Street Cambridge MA 02138 USA; ^16^ Cooperative Institute for Mesoscale Meteorological Studies University of Oklahoma National Weather Center Suite 2100 120 David L. Boren Blvd Norman OK 73072 USA; ^17^ Fundación Con‐Vida Cr68 A 46 A‐77 Medellín Medellín Colombia; ^18^ Museo de Historia Natural Noel Kempff Mercado Universidad Autonoma Gabriel Rene Moreno Casilla 2489, Av. Irala 565 Santa Cruz Bolivia; ^19^ UNELLEZ‐Guanare, Programa de Ciencias del Agro y el Mar, Herbario Universitario (PORT) Mesa de Cavacas Estado Portuguesa 3350 Venezuela; ^20^ Department of Biological Sciences International Center for Tropical Botany (ICTB) Florida International University 112200 SW 8th Street, OE 167 Miami FL 33199 USA; ^21^ Universidade Federal do Acre Campus de Cruzeiro do Sul Rio Branco Brazil; ^22^ INRA UMR 1137 “Ecologie et Ecophysiologie Forestiere” 54280 Champenoux France; ^23^ Tropenbos International PO Box 232 6700 AE Wageningen The Netherlands; ^24^ Université Paul Sabatier CNRS UMR 5174 Evolution et Diversité Biologique bâtiment 4R1 31062 Toulouse France; ^25^ Jardín Botánico de Medellín Joaquín Antonio Uribe Calle 73 # 51 D 14 Medellín Cartagena Colombia; ^26^ Andes to Amazon Biodiversity Program Puerto Maldonado Madre de Dios Perú; ^27^ Centro de Geociencias Universidade Federal do Para CEP 66017‐970 Belem Para Brazil; ^28^ Department of Anthropology University of Texas at Austin SAC Room 5.150 2201 Speedway Stop C3200 Austin TX 78712 USA; ^29^ Museu Paraense Emilio Goeldi Av. Magalhães Barata, 376 ‐ São Braz CEP: 66040‐170 Belém PA Brazil; ^30^ Instituto de Investigaciones de la Amazonía Peruana Av. José Quiñones km 2.5 Iquitos Perú; ^31^ World Wildlife Fund 1250 24th St NW Washington DC 20037 USA; ^32^ Centre for Tropical Environmental and Sustainability Science (TESS) and College of Marine and Environmental Sciences James Cook University Cairns Qld 4878 Australia; ^33^ Instituto Boliviano de Investigación Forestal C.P. 6201 Santa Cruz de la Sierra Bolivia; ^34^ Environmental Science and Policy Department and the Department of Public and International Affairs at George Mason University (GMU) 3351 Fairfax Drive Arlington Washington DC VA 22201 USA; ^35^ Environmental Change Institute School of Geography and the Environment University of Oxford South Parks Road Oxford OX1 3QY UK; ^36^ Universidade do Estado de Mato Grosso Campus de Nova Xavantina Caixa Postal 08 CEP 78.690‐000 Nova Xavantina MT Brazil; ^37^ Escuela de Ciencias Forestales (ESFOR) Av. Final Atahuallpa s/n Casilla 447 Cochabamba Bolivia; ^38^ Facultad de Ingeniería Ambiental Universidad Estatal Amazónica Paso lateral km 2 1/2 via Napo Puyo Pastaza Ecuador; ^39^ Universidad Autonoma del Beni Campus Universitario Av. Ejército Nacional, final Riberalta Beni Bolivia; ^40^ Forest Ecology and Forest Management Group Wageningen University PO Box 47 Wageningen 6700 AA The Netherlands; ^41^ Center for Tropical Conservation Duke University Box 90381 Durham NC 27708 USA; ^42^ Doctorado Instituto de Ciencias Naturales Universidad Nacional de Colombia Bogotá Colombia; ^43^ Instituto de Investigaciones para el Desarrollo Forestal Universidad de Los Andes Avenida Principal Chorros de Milla Campus Universitario Forestal Edificio Principal Mérida Venezuela; ^44^ Iwokrama International Centre for Rainforest Conservation and Development 77 High Street Kingston Georgetown Guyana; ^45^ Museu Universitário Universidade Federal do Acre Rio Branco AC 69910‐900 Brazil; ^46^ Institute of Biological and Health Sciences Federal University of Alagoas Av. Lourival Melo Mota s/n Tabuleiro do Martins, Maceió AL 57072‐900 Brazil; ^47^ Naturalis Biodiversity Center PO Box 9517 2300 RA Leiden The Netherlands; ^48^ School of Geography University of Nottingham Nottingham NG7 2RD UK; ^49^ Centro de Investigación y Promoción del Campesinado, regional Norte Amazónico C/Nicanor Gonzalo Salvatierra N° 362 Casilla 16 Riberalta Bolivia; ^50^ Universidad Autónoma del Beni Avenida 6 de Agosto N° 64 Riberalta Bolivia

**Keywords:** allometry, carbon, dynamic global vegetation model, forest plots, productivity, tropical forest

## Abstract

Understanding the processes that determine above‐ground biomass (AGB) in Amazonian forests is important for predicting the sensitivity of these ecosystems to environmental change and for designing and evaluating dynamic global vegetation models (DGVMs). AGB is determined by inputs from woody productivity [woody net primary productivity (NPP)] and the rate at which carbon is lost through tree mortality. Here, we test whether two direct metrics of tree mortality (the absolute rate of woody biomass loss and the rate of stem mortality) and/or woody NPP, control variation in AGB among 167 plots in intact forest across Amazonia. We then compare these relationships and the observed variation in AGB and woody NPP with the predictions of four DGVMs. The observations show that stem mortality rates, rather than absolute rates of woody biomass loss, are the most important predictor of AGB, which is consistent with the importance of stand size structure for determining spatial variation in AGB. The relationship between stem mortality rates and AGB varies among different regions of Amazonia, indicating that variation in wood density and height/diameter relationships also influences AGB. In contrast to previous findings, we find that woody NPP is not correlated with stem mortality rates and is weakly positively correlated with AGB. Across the four models, basin‐wide average AGB is similar to the mean of the observations. However, the models consistently overestimate woody NPP and poorly represent the spatial patterns of both AGB and woody NPP estimated using plot data. In marked contrast to the observations, DGVMs typically show strong positive relationships between woody NPP and AGB. Resolving these differences will require incorporating forest size structure, mechanistic models of stem mortality and variation in functional composition in DGVMs.

## Introduction

Tropical forests are the most carbon‐rich and productive of all forest biomes (Pan *et al*., 2011). The Amazon basin in particular comprises approximately 50% of the world's tropical forests, and therefore, any perturbations to this ecosystem will have important feedbacks on both carbon cycling and climate worldwide (Zhao & Running, 2010; Wang *et al*., 2014). It is therefore important that we understand the processes that determine current patterns of carbon storage and cycling to predict how the productivity and carbon stores of these forests will respond to changing environmental conditions.

Our knowledge of the sensitivity of rainforest ecosystems to environmental change is based on three sources. Firstly, observational data from networks of permanent plots, flux towers, remote sensing and aircraft measurements of greenhouse gas concentrations have demonstrated the sensitivity of these ecosystems to environmental change, particularly in response to drought (e.g. Phillips *et al*., 2009; Restrepo‐Coupe *et al*., 2013; Gatti *et al*., 2014). Secondly, experimental manipulations of water stress have probed the mechanisms behind these responses (e.g. Nepstad *et al*., 2007; da Costa *et al*., 2010; Meir *et al*., 2015; Rowland *et al*., 2015). Thirdly, process‐based ecosystem models, especially dynamic global vegetation models (DGVMs), have been used to explore the future sensitivity of Amazon vegetation to increasing temperatures, carbon dioxide concentrations and water stress (e.g. Galbraith *et al*., 2010). Coupled with climate models, DGVMs have highlighted the sensitivity (Cox *et al*., 2004), and more recently, the resilience (Rammig *et al*., 2010; Huntingford *et al*., 2013) of Amazonian forests to environmental change. However, observations of above‐ground biomass (AGB, Mg C ha^−1^) and woody productivity (the amount of net primary productivity (NPP) allocated to above‐ground woody growth: *W*_P_, Mg C ha^−1^ yr^−1^) are still little used to parameterize and evaluate DGVMs (e.g. Delbart *et al*., 2010; Castanho *et al*., 2013), despite substantial progress increasing the spatial distribution of such *in situ* observations (e.g. Feldpausch *et al*., 2011; Quesada *et al*., 2012; Mitchard *et al*., 2014). Integrating the insights from such observational studies into the design, calibration and validation of DGVMs would enhance our ability to make convincing predictions of the future of tropical carbon.

Observational data can either be used to evaluate the outputs of models, or more fundamentally, calibrate and inform the processes that models should aim to include. For example, networks of inventory plots have revealed strong differences in AGB among *terra firme* forests in north‐east and south‐western Amazonia (Baker *et al*., 2004; Malhi *et al*., 2006; Baraloto *et al*., 2011; Quesada *et al*., 2012; Mitchard *et al*., 2014). Such observations have been used to evaluate the predictions of Amazonian forest biomass from both remote sensing (e.g. Mitchard *et al*., 2014) and DGVM studies (e.g. Castanho *et al*., 2013). These field observations also yield information about the processes that drive variation in above‐ground carbon stocks, which can also be used to evaluate and calibrate DGVMs. For example, the paradigm to emerge from previous analysis of plot data in Amazonia is that there is a positive association between woody NPP and stem mortality rates, linked to a reduction in AGB (Baker *et al*., 2004; Malhi *et al*., 2004; Quesada *et al*., 2012). This finding has been used to evaluate the architecture and outputs of DVGMs (Negrón‐Juárez *et al*., 2015) and has stimulated attempts to make direct links between mortality and woody NPP in these models (Delbart *et al*., 2010; Castanho *et al*., 2013).

More generally, observational data are valuable for informing how the fundamental processes that influence AGB should be included in vegetation models. For example, the residence time of woody biomass, *τ*_w_ (years), is often used as a measure of mortality in DGVMs and is defined for a forest at steady state as:

(1) $$\tau_{w}=\frac{\text{AGB}}{W_{P}}.$$

This parameter varies almost sixfold among tropical forest plots (Galbraith *et al*., 2013). However, surprisingly, in several commonly used vegetation models, this parameter is constant; Galbraith *et al*. (2013) found that 21 of the 27 vegetation models they compared use single, fixed values for this parameter. In addition, observational data suggest that the ultimate cause of variation in tree mortality, *W*_P_ and hence AGB is variation in edaphic properties (Quesada *et al*., 2012). Quesada *et al*. (2012) found that spatial differences in *W*_P_ correlated most strongly with total soil phosphorus, whereas stem mortality rates correlated with a soil physical structure index which combined soil depth, texture, topography and anoxia. Most DGVMs, however, only include very limited feedbacks between vegetation and edaphic properties. Soil properties such as texture are mainly implemented into DGVMs to parameterize hydraulic processes (e.g. Marthews *et al*., 2014) and soil structure and nutrient content are rarely considered for other processes such as stem mortality.

Overall, the aim of this study is to compare how variation in *W*_P_ and mortality control variation in AGB in Amazonia using both field observations and four DGVMs, to inform the future development of vegetation models. In terms of the analysis of observations, we build on previous work (e.g. Baker *et al*., 2004; Malhi *et al*., 2004, 2015) in two ways. Firstly, we compare patterns of AGB with variation in two direct measurements of mortality from each plot: the absolute, stand‐level rate of woody biomass loss (*W*_L_; Mg C ha^−1^ yr^−1^) and the rate of stem mortality (μ; % yr^−1^). Previous studies have used *τ*_w_ to examine how mortality influences AGB (e.g. Malhi *et al*., 2004, 2015; Galbraith *et al*., 2013). However, although *τ*_w_ is a useful parameter in the context of vegetation modelling and to partition ecosystem carbon fluxes, its dependency on AGB (see Eqn (1)) means that this term is not an independent control of AGB stocks: it is inevitable that AGB is inversely related to *τ*_w_. In addition, as *τ*_w_ is defined for a forest at steady state, it cannot be easily related to specific short‐term processes, such as droughts, which ultimately cause tree mortality. Here, we therefore test the sensitivity of AGB to direct independent measures of both stand‐level and stem‐level variation in mortality rates, as these measures may ultimately provide a more appropriate basis for modelling mortality in DGVMs.

**Figure 1:**
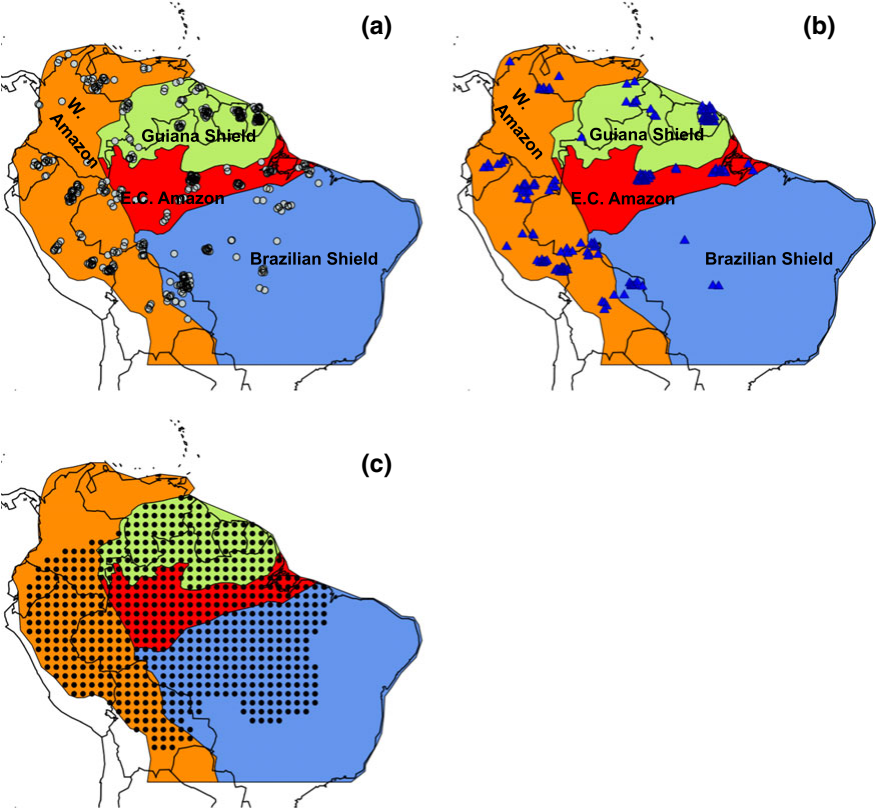
Location of plots used to calculate (a) above‐ground woody biomass, (b) above‐ground woody productivity and stem and biomass‐based mortality and (c) the position of the kriged 1° map grid cells. The Amazon basin including forests on the Guiana Shield is split into regions (shown by different colours) that are defined in Feldpausch *et al*. (2011). Plot locations are not geographically exact but are offset slightly to improve the visualization of plots which are in very close proximity to each other.

Secondly, we greatly extend the spatial coverage of observations. The first large‐scale studies of Amazon forest dynamics (Baker *et al*., 2004; Malhi *et al*., 2004; Phillips *et al*., 2004) focused on the western, and central and eastern portions of the basin, but included few data from forests on the Guiana and Brazilian Shields (Fig. 1). These areas, however, have distinctive soils, climate, forest structure and species composition (e.g. ter Steege *et al*., 2006; Feldpausch *et al*., 2011). Here, we use data from these regions to test whether the paradigm of a positive association between woody NPP and stem mortality rates, linked to a reduction in AGB, is found across the full range of South American lowland moist tropical forests.

In terms of the analysis of the DGVMs, we aim firstly to establish the reliability of land vegetation simulation for the Amazon basin by comparison of modelling results with kriged maps of field observations of *W*_P_, mortality and AGB that illustrate the major patterns of variation in these variables. We then test how well the four DGVMs capture these spatial patterns and the overall magnitude of AGB and *W*_P_. Finally, we explore the relationships between simulated AGB, *W*_P_ and *τ*_w_. By comparing our findings from the analysis of the observations and simulation results, we conclude by making recommendations for model developments and data collection that will improve our ability to model Amazonian vegetation carbon stocks.

## Materials and methods

### Plot observations

We used tree inventory data from permanent sample plots located throughout Amazonia compiled as part of the RAINFOR and TEAM networks to estimate stocks (AGB) and fluxes of carbon (woody NPP, stem and biomass mortality) within Amazonian forest stands (Fig. 1). For analysis of AGB, we used the data for the 413 plots analysed by Mitchard *et al*. (2014) (Fig. 1a). For properties which can only be calculated by observing change over time and thus require more than one census, plots in intact, moist, lowland (<1000 m asl) forest were chosen which had a minimum total monitoring period of 2 years between 1995 and 2009 inclusive. Data for 167 plots that met these criteria for analysis of dynamic properties were downloaded from ForestPlots.net (Lopez‐Gonzalez *et al*., 2011, 2012; Johnson *et al*., 2016; Fig. 1b and Table S1) and the TEAM website (http://www.teamnetwork.org/data/query; data set identifier codes 20130415013221_3991 and 20130405063033_1587). For this data set, mean plot size is 1.09 ha, the mean date of the first census is 2000.2 and the mean date of the final census is 2008.5. Mean census interval length is 3.70 years and plot mean total monitoring period is 8.3 years. Most of the plots were monitored for most of the time period: on average, 76% of plots were being monitored in any given year from 2000–2008 (Fig. S1). All trees with a diameter at breast height (dbh) greater than 10 cm were included in the analyses.

Plots were classified into four regions of lowland moist forest defined by the nature and geological age of the soil substrate (Fig. 1; Feldpausch *et al*., 2011). The soils and forests of the Guiana and Brazilian Shields have developed on old, Cretaceous, crystalline substrates, whereas the forests of Western Amazonia are underlain by younger Andean substrates and Miocene deposits (Irion, 1978; Quesada *et al*., 2010; Higgins *et al*., 2011). East‐central Amazonia contains reworked sediments derived from the other three regions that have undergone almost continuous weathering for more than 20 million years, leading to very nutrient poor soils (Irion, 1978; Quesada *et al*., 2010). Previous comparative studies have noted substantial differences in forest dynamics between Western and East‐central Amazonia (Baker *et al*., 2004, 2014; Quesada *et al*., 2012), but largely excluded forests on the Guiana and Brazilian Shields. This classification therefore allows us to test the impact of including these distinctive forests on Amazon‐wide patterns of forest dynamics.

### Above‐ground biomass

For AGB values, we used the data set presented by Mitchard *et al*. (2014) and Lopez‐Gonzalez *et al*. (2014). In brief, for this data set, the AGB (Mg DW ha^−1^) of each plot was calculated using the Chave *et al*. (2005) moist forest allometric equation which includes measurements of diameter, wood density and height:

(2) $$\text{AGB}=\frac{\sum_{1}^{n}\left(0.0509\rho D^{2}H\right)}{1000},$$

where *D* is stem diameter (cm), *ρ* is stem wood density (g cm^−3^), *H* is stem height (m) and *n* is the number of trees in the stand. We retained the use of this biomass equation for this study, instead of using the recent biomass equation of Chave *et al*. (2014), to provide estimates of *W*_P_ that are consistent with Mitchard *et al*. (2014). Estimates of AGB for moist tropical forests are in fact similar using either equation (Chave *et al*., 2014). The height of each tree was estimated from tree diameter using a height‐diameter Weibull equation with different coefficients for each region, based on field‐measured, height‐diameter relationships (Feldpausch *et al*., 2011). We used this method to estimate tree height, rather than predicting height on the basis of climate as in Chave *et al*. (2014), because among moist forests in Amazonia, the principal variation in height/diameter allometry is due to the contrast between the particularly tall‐statured forests on the Guiana Shield and shorter‐statured forest in other regions (Feldpausch *et al*., 2011). This difference is related to the unique species composition of forests on the Guiana Shield rather than variation in climate (Feldpausch *et al*., 2011). The wood density of each tree was assigned on a taxonomic basis from the pan‐tropical database of Zanne *et al*. (2009) and Chave *et al*. (2009), following Baker *et al*. (2004). Mean plot wood density values were used when taxonomic information was missing for individual trees.

To estimate total above‐ground woody biomass, we assumed that carbon is 50% of total dry biomass (Penman *et al*., 2003) and to account for the unmeasured, small trees (<10 cm), we added an additional 6.2% of carbon to each of the plots, following Malhi *et al*. (2006). We do not include the unknown contributions from lianas, epiphytes, necromass, shrubs and herbs.

### Mortality and productivity

Stem mortality rates were calculated as the exponential mortality coefficient μ [% yr^−1^; Sheil & May (1996)]:

(3) $$\mu =\frac{\text{ln}\;\left(n_{0}\right)-\text{ln}\;\left(n_{0}-n_{d}\right)}{t}\times 100,$$

where *n*_*0*_ is the number of stems at the start of the census interval, *n*_d_ is the number of stems that die in the interval and *t* is the census interval length. As estimates of mortality rates in heterogeneous populations are influenced by the census interval, we standardized our estimates of μ to comparable census intervals using the equation of Lewis *et al*. (2004). We calculated corrected values of μ for each census interval for each plot in the data set, and calculated average values of μ per plot, weighted by the census interval length.

Total NPP cannot be calculated from tree inventories as this includes both the growth of the stem as well as litterfall and root production which has only been measured at a relatively small number of Amazonian sites (Malhi *et al*., 2015). Therefore, we are restricted to calculating *W*_P_, which can be calculated from repeated censuses of tree diameters within inventory plots. Comparable output can be obtained from vegetation models as DGVMs typically partition total above‐ground NPP into different carbon pools using various carbon allocation algorithms, ranging from fixed coefficients (e.g. INLAND) to approaches based on resource limitation (e.g. ORCHIDEE). For comparison with measurement data, we used the fraction of simulated above‐ground NPP that the models allocate to woody growth. Both the observed measurements and models exclude the contribution to *W*_P_ that is made by the loss and regrowth of large woody branches. This component is approximately 1 Mg C ha^−1^ a^−1^ in Amazonian forests or 10% of above‐ground NPP (Malhi *et al*., 2009). *W*_L_ was calculated as the sum of the biomass of all trees that died within a given census interval.

Estimates of *W*_P_ and *W*_L_ are influenced by the census interval over which they are calculated, because more trees will recruit and die without being recorded during longer census intervals (Talbot *et al*., 2014). We followed the methods of Talbot *et al*. (2014) for calculating *W*_P_ with forest inventory data to correct for this bias (Supporting information, Appendix S1). Thus, we calculated *W*_P_ as the sum of (i) the growth of trees that survive the census period, and the estimated growth of (ii) trees that died during the census interval, prior to their death, (iii) trees which recruited within the interval, and (iv) trees that both recruited and died during the census interval. Similarly, to calculate *W*_L,_ we summed the biomass of trees that die within a census interval with components (ii) and (iv) above. We calculated corrected values of *W*_P_ and *W*_L_ for each census interval for each plot in the data set, and calculated average values per plot, weighted by census interval length.

### Analysis of observational data

The current paradigm for Amazonian forests suggests that *W*_P_ and μ are positively correlated and that both correlate negatively with AGB (Malhi *et al*., 2002; Quesada *et al*., 2012). We tested whether these relationships are supported by the data from across South American tropical lowland moist forest, including plots from the Guiana and Brazilian Shield. Firstly, we explored whether different regions have distinctive patterns of carbon cycling by comparing *W*_P_ *, W*_L_, μ and AGB among the four regions using anova. Secondly, we explored the relationships between these terms using generalized least squares regression. We tested whether *W*_P_ and either *W*_L_ or μ were significantly related to AGB and whether these relationships differed among the four regions. We accounted for spatial autocorrelation by specifying a Gaussian spatial correlation structure, which is consistent with the shape of the semivariograms for these forest properties across the plot network (Fig. S2). Stem mortality rates and absolute rates of woody biomass loss were log‐transformed prior to analysis to ensure the residuals were normally distributed. Model evaluation was performed on the basis of Akaike information criterion (AIC) values. Analyses were carried out using the *nlme* package in r (R Development Core Team, 2012; Pinheiro *et al*., 2016).

### Model simulations and comparison with observations

We tested how well a range of DGVMs perform for Amazonia by comparing observed AGB, *W*_P_ and *τ*_w_ to the output from four DGVMs. The DGVMs included in this study are the joint uk land environment simulator (jules), v. 2.1. (Best *et al*., 2011; Clark *et al*., 2011), the Lund‐Potsdam‐Jena DGVM for managed Land (LPJmL; Sitch *et al*., 2003; Gerten *et al*., 2004; Bondeau *et al*., 2007), the INtegrated model of LAND surface processes (INLAND) model (a development of the IBIS model, Kucharik *et al*., 2000) and the Organising Carbon and Hydrology In Dynamic EcosystEms (ORCHIDEE) model (Krinner *et al*., 2005). A brief description of each of the four models and how output data are derived is included in the supplementary information (Appendix S2). The models each followed the standardized Moore Foundation Andes‐Amazon Initiative (AAI) modelling protocol (Zhang *et al*., 2015). The simulated region spanned 88°W to 34°W and 13°N to 25°S. Simulations from each model included a spin‐up period from bare ground of up to 500 years with pre‐industrial atmospheric CO_2_ (278 ppm). The models were then forced by recycling 39 year, 1° spatial resolution, bias‐corrected NCEP meteorological data (Sheffield *et al*., 2006) for 1715–2008 with increasing CO_2_ concentrations, as in Zhang *et al*. (2015). Figure S3 shows the spatial distribution of mean meteorological variables for 2000–2008 across the Amazon basin. As well as precipitation, temperature and short‐wave radiation we also show maximum cumulative water deficit (MWD), calculated from monthly precipitation values to indicate drought severity across the basin, as in Aragao *et al*. (2007). The time period of model output is 2000–2008.

To compare simulated woody NPP with observed *W*_P_, corrections were applied to the simulated total woody NPP to calculate above‐ground woody NPP only, by assuming a below‐ground to above‐ground allocation ratio of 0.21 (Malhi *et al*., 2009). In the case of JULES, only a fraction of the NPP is allocated to biomass growth, as the remainder is allocated to ‘spreading’ of vegetated area – an increase in the fraction of grid cell cover (Cox, 2001). To facilitate comparison with observations and other models, we therefore rescaled *W*_P_ from JULES, retaining the relative allocation to wood but assuming that all of the NPP was used for growth.

We compared model outputs to kriged maps of AGB, *W*_P_ and mortality to understand how well the DGVMs captured the major differences in AGB, *W*_P_ and mortality across the basin. The forest properties were mapped onto a region defined as Amazonia *sensu stricto* (Eva *et al*., 2005) which is divided into 1° by 1° longitude–latitude grid cells (Fig. 1c). Model output was provided for the same grid. The kriged maps were created using ordinary kriging with the *gstat* package in r (Pebesma, 2004). To assess the predictive ability of the kriging method, we performed a leave‐one‐out cross‐validation technique. This involves leaving one site out in turn and performing the kriging using the rest of the observations. The kriging prediction for this location was then compared with the observation. Results from the cross‐validation demonstrate that there was no spatial bias in the kriging method (Fig. S4). There was also no tendency for the kriging to overestimate or underestimate values for the whole basin. However, the kriging method was not able to capture the few locations with very high mortality values (Fig. S5). This problem is common to any interpolation method which is effectively averaging observed values. The median percentage bias between the leave‐one‐out cross‐validation and the measured plot values was 13.6%, 12.7% and 23.0% for AGB, *W*_P_ and stem mortality rate respectively.

We do not intend the kriged maps to be a detailed, accurate description of Amazon forest properties: ecological patterns are a mix of smooth gradients (e.g. related to climate) and more abrupt boundaries (e.g. related to edaphic properties) that cannot be shown using these methods. Rather, we intend these maps as broad scale tools to provide a means of evaluating the performance of the vegetation models.

Finally, we compared how well the DGVMs captured the mean and variability in AGB, *W*_P_ and *τ*_w_ (calculated using average values for *W*_P_ and AGB across all grid cells for 2000–2008 from model outputs using Eqn (1)) for grid cells where there is observational data, and contrast the controls on AGB between observations and models in terms of *W*_P_ and mortality. We acknowledge that the models will predict a small increase in *W*_P_ over the time period of study due to CO_2_ fertilization (~0.35 Mg C ha^−1^ a^−1^; Lewis *et al*., 2009). However, the effect of this process on estimates of *τ*_w_ is small.

## Results

### Observed links between woody biomass, mortality and productivity

**Figure 2:**
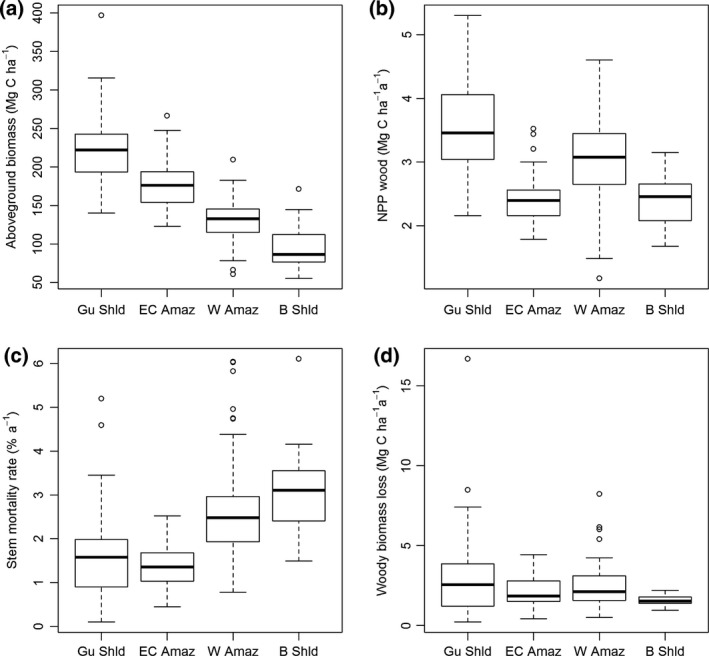
Boxplots of plot measurements of (a) above‐ground biomass, (b) above‐ground woody productivity, (c) stem mortality rates and (d) absolute rates of woody biomass loss in four regions of Amazonia. Gu Shld = Guiana Shield, EC Amaz = East Central Amazon, W Amaz = Western Amazon, B Shld = Brazilian Shield.

There is a strong variation in AGB (*F*_3,163_ = 72.1, *P* < 0.001), μ (*F*_3,163_ = 23.6, *P* < 0.001) and *W*_P_ (*F*_3,163_ = 22.7, *P* < 0.001) among the four regions, but not *W*_L_ (*F*_3,163_ = 1.49, ns; Table 1, Fig. 2). Forests on the Guiana Shield are characterized by the highest AGB of all Amazonian forests, associated with low stem mortality rates and high *W*_P_ (Fig. 2a–c). East‐central Amazon forests also have comparatively high AGB and similar, very low stem mortality rates. However, *W*_P_ is lower in these sites (Fig. 2b). Compared with these regions, forests in the western Amazon and on the Brazilian Shield have lower AGB. However, the lower biomass in these two regions is associated with different patterns in *W*_P_. In the western Amazon, the lower biomass values are associated with high *W*_P_ (Fig. 2a–c). In contrast, the particularly low biomass forests of the Brazilian Shield have high rates of stem mortality and low *W*_P_ (Fig. 2a–c).

**Table 1:** Observed forest properties (mean ± SE) calculated from plot data for each region of Amazonia

	Basin	Guiana Shield	East‐central Amazon	Western Amazon	Brazilian Shield
Mean above‐ground biomass (Mg C ha^−1^)	153.48 ± 2.82 *n* = 413	211.91 ± 5.03 *n* = 110	167.64 ± 4.95 *n* = 78	126.26 ± 2.38 *n* = 149	107.73 ± 4.48 *n* = 76
Mean above‐ground woody productivity (Mg C ha^−1^ yr^−1^)	2.97 ± 0.06 *n* = 167	3.51 ± 0.13 *n* = 41	2.41 ± 0.07 *n* = 37	3.06 ± 0.07 *n* = 76	2.40 ± 0.15 *n* = 13
Stem‐based mortality rate (% yr^−1^)	1.96 ± 0.08 *n* = 167	1.66 ± 0.16 *n* = 41	1.38 ± 0.08 *n* = 37	2.62 ± 0.12 *n* = 76	3.19 ± 0.38 *n* = 13
Mean above‐ground biomass losses (Mg C ha^−1^ yr^−1^)	2.46 ± 0.13 *n* = 167	3.06 ± 0.44 *n* = 41	2.12 ± 0.16 *n* = 37	2.43 ± 0.15 *n* = 76	1.57 ± 0.12 *n* = 13
Mean wood density (g cm^−3^)	0.63 ± 0.00 *n* = 413	0.69 ± 0.00 *n* = 110	0.67 ± 0.01 *n* = 78	0.58 ± 0.00 *n* = 149	0.61 ± 0.01 *n* = 76
Basal area (m^2^ ha^−1^)	26.64 ± 5.53 *n* = 413	29.10 ± 0.49 *n* = 110	28.24 ± 0.51 *n* = 78	25.98 ± 0.41 *n* = 149	22.73 ± 0.66 *n* = 76

Analysis of the relationships using generalized least squares allows the relative importance of *W*_P_ and μ for determining AGB to be explored in more detail. Stem mortality rate is the key parameter that controls variation in AGB (Table 2, Fig. 4c). This relationship between AGB and stem mortality rates is not because there is a correlation between AGB and stem number, as these two variables are unrelated (Fig. S6). In contrast, the alternative measure of mortality, *W*_L_, is not related to AGB (Fig. 4b): all models including stem mortality rates, rather than *W*_L_, show substantially better fit and lower AIC values (Table 2).

**Table 2:** Generalized least squares models relating AGB to variation in (A) above‐ground woody productivity (*W*_P_), stem mortality rates (μ) or rates of woody biomass loss (*W*_L_); (B) μ and *W*_P_; (C) *W*_L_ and *W*_P_ among 167 plots across four regions of Amazonia. Models incorporated region as an additional factor and interactions as appropriate. Terms for mortality were log‐transformed before analysis. All models incorporated a Gaussian spatial error correlation structure to account for spatial autocorrelation. The model with the strongest support is highlighted in bold; this model was used to quantify the relationships in Fig. 3

Model	Terms	Interactions	Log likelihood	AIC	Pseudo *r* squared
A. Including either mortality or growth					
1	μ*,* Region		−813.7	1643.3	0.65
2	*W*_L_ *,* Region		−830.1	1676.3	0.57
3	*W*_P_, Region		−829.3	1674.5	0.58
B. Including *W*_P_ and μ as mortality term					
4	*W*_P_, μ*,* Region		−810.8	1639.6	0.66
**5**	***W***_**P**_ **, μ** ***,*** **Region**	**μ** * *×* * **Region**	−**805.0**	**1634.0**	**0.68**
6	*W*_P_, μ*,* Region	*W*_P_ * *×* *Region	−808.8	1641.6	0.67
C. Including *W*_P_ and *W*_L_ as mortality term					
7	*W*_P_, *W*_L_ *,* Region		−829.0	1676.1	0.58
8	*W*_P_, *W*_L_ *,* Region	*W*_L_ * *×* *Region	−826.7	1677.4	0.59
9	*W*_P_, *W*_L_ *,* Region	*W*_P_ * *×* *Region	−826.6	1677.2	0.59

AGB, above‐ground biomass.

The effect of stem mortality rate on AGB also differs among regions (Fig. 4c). For example, for a stem mortality rate of 1.5% yr^−1^, forests on the Guiana Shield store approximately 75% more carbon as (above‐ground) wood than forests on the Brazilian Shield (Fig. 4c). In addition, the strength of the relationship between AGB and stem mortality rates varies among regions: the slope of this relationship is comparatively shallow among the plots in western Amazonia (Fig. 4c). Finally, *W*_P_ is significantly positively correlated with variation in AGB, although the relationship is weak (Table 2, Fig. 4a).

### Model projections and comparison with observations

The comparisons of simulated AGB and above‐ground *W*_P_ reveal considerable differences both between the individual models and between the models and observations (Table 3, Figs 5, 6, S7 and S8). For the whole of the Amazon basin, mean AGB is highest for ORCHIDEE, and lowest for INLAND; in contrast, woody NPP is highest for LPJmL and lowest for JULES (Table 3). Compared with the plots, different models over‐ and underestimate mean AGB (Table 3). However, the model ensemble mean AGB value (163.87 Mg C ha^−1^) is close to the observed mean (153.48 Mg C ha^−1^). In contrast, all models overestimate above‐ground *W*_P_ compared with the mean for the plots, by between 36% (JULES) and 234% (LPJmL; Table 3, Fig. 5). Variation in *τ*_w_ inevitably reflects the variation in mean AGB and woody NPP with average values for ORCHIDEE and JULES (27.9 and 33.2 years) approximately twice the values for INLAND and LPJmL (16.7 and 17.5 years).

**Table 3:** Basin mean values, standard errors and root mean square error (RMSE) for above‐ground wood biomass (AGB; Mg C ha^−1^) and above‐ground woody net primary productivity (woody NPP; Mg C ha^−1^ yr^−1^) from the plot observations and mean values from four DGVMs for the plot locations. A below‐ground to above‐ground allocation ratio of 0.21 is applied to the DGVM values to convert from total NPP wood to above‐ground woody NPP

Model	AGB (Obs mean = 153.48)				*W*_P_ (Obs mean = 2.97)			
	AGB wood				AG NPP wood			
	ORCHIDEE	JULES	INLAND	LPJmL	ORCHIDEE	JULES	INLAND	LPJmL
Model mean	218.00 ± 3.16	137.93 ± 2.09	125.43 ± 1.35	174.10 ± 2.89	7.80 ± 0.10	4.05 ± 0.09	7.46 ± 0.11	9.92 ± 0.10
RMSE	91.84	76.98	61.36	73.65	5.00	1.89	4.73	7.06

NPP, net primary productivity; DGVMs, dynamic global vegetation models.

There are considerable differences between the observations and the predictions across the four models in the spatial variability of AGB and *W*_P_ (Figs 5, 6 and S7). JULES and INLAND both simulate very little spatial heterogeneity in AGB in the Amazon basin, in contrast to the strong pattern in the observations: compared with the observations, they simulate a very narrow range of AGB values and underestimate both the AGB of the Guiana Shield and the basin as a whole (Table 3, Fig. 5c, e). LPJmL and ORCHIDEE display greater variability in their predictions of AGB (Fig. 5g, i). However, LPJmL predicts highest AGB in the north‐west of the basin in contrast to the observations (Fig. 5i). ORCHIDEE is the only model that provides a reasonable match with the spatial patterns in the observations, but this model still overestimates AGB for most of the basin compared with the plot observations (Table 3, Fig. 5g).

In terms of *W*_P_, LPJmL (Fig. 5j) is the only model that captures the higher observed values in the Guiana Shield and Western Amazon compared with the Brazilian Shield and East‐central Amazon (Fig. 5b). In contrast, INLAND, ORCHIDEE and JULES simulate very little variability in *W*_P_ across the majority of basin (Fig. 5d, f, h).

For all models, the spatial variation in *τ*_w_ is similar to that of AGB (Fig. 6). LPJmL demonstrates the greatest spatial variation in residence times with the highest values found in the north‐west of the basin (Fig. 6). JULES and INLAND display little variation in *τ*_w_ across the basin. Overall, JULES, LPJmL and INLAND display a much stronger positive relationship between woody NPP and AGB (Fig. 7) than seen in the observations (Fig. 4a), although the form of this relationship varies. In contrast, the relationship predicted by ORCHIDEE matches the variability and form of the relationship between woody NPP and AGB from the plot data comparatively well (Fig. 7).

Simulated AGB and *W*_P_ from all four models show strong relationships with climatological drivers. Correlations between *W*_P_ and precipitation are particularly strong for INLAND and LPJmL and all models apart from JULES exhibit strong correlations between rainfall and AGB (Fig. S9). Weaker correlations are observed between temperature and short‐wave radiation and simulated *W*_P_ and AGB (Fig. S10).

## Discussion

### Understanding spatial variation in the AGB of Amazon forests

Overall, our results extend and enrich the original paradigm concerning the controls on forest dynamics in Amazonia. The previous paradigm described correlated west to east gradients in *W*_P_, stem mortality rates and AGB across the Amazon basin, maintained by a soil‐mediated, positive feedback mechanism (Malhi *et al*., 2004; Quesada *et al*., 2012). Our findings agree that variation in mortality is the key driver of variation in AGB across Amazonian forests (Table 2, Fig. 4). However, our results modify the current paradigm about variation in forest dynamics in Amazonia in four important ways.

**Figure 3:**
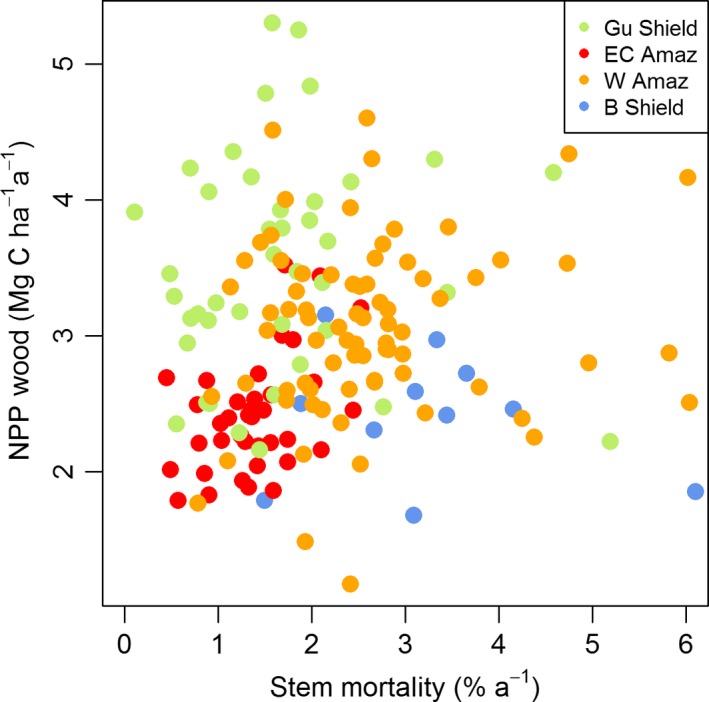
Relationship between woody net primary productivity (NPP) and stem mortality rates for 167 forest plots in four regions of Amazonia.

Firstly, the plot data demonstrate that there is no correlation between *W*_P_ (above‐ground woody productivity) and stem mortality rates with the new, broader data set: they vary independently (Fig. 3). Previous studies have strongly focused on western Amazonia and some East‐central Amazon sites. However, the inclusion of data from the Guiana Shield in particular demonstrates that low stem mortality rates can also be associated with high *W*_P_ (Fig. 3).

**Figure 4:**
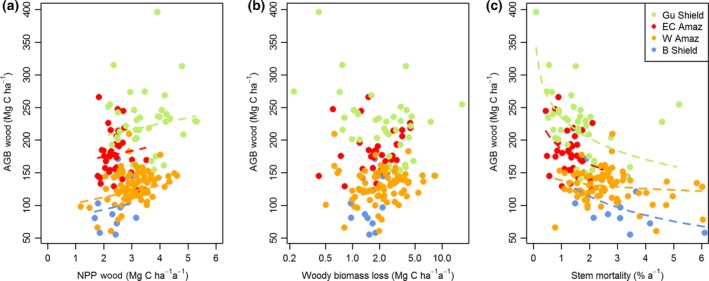
Relationships between AGB and (a) woody NPP, (b) absolute rates of woody biomass loss and (c) stem mortality rates for 167 forest plots in four regions of Amazonia. Lines relate to significant relationships as given by final statistical model in Table 3. NPP, net primary productivity; AGB, above‐ground biomass.

Secondly, our results demonstrate that variation in stem mortality rates, rather than absolute rates of carbon loss, is the key aspect of mortality that determines variation in AGB. The lack of correlation between AGB and absolute rates of biomass loss (Fig. 4b) is somewhat surprising: for a forest stand at approximately steady state, we might expect this relationship to at least mirror the weak correlation between AGB and stand *W*_P_ (Fig. 4a). This result may be because estimates of absolute AGB loss are subject to greater sampling error than *W*_P_ due to stochastic variation in tree mortality (e.g. see wide variation in values on the *x* axis of Fig. 4b). Sampling over longer time intervals may reveal stronger correlations between absolute rates of biomass loss and AGB.

In contrast to these patterns for absolute rates of loss of biomass, there are strong relationships between stem mortality rates and AGB (Fig. 4c). This result suggests that variation in the numbers and diameters of trees that die in different locations is a key control on AGB: high rates of stand‐level biomass loss and *W*_P_ can be associated with high AGB if stem mortality rates are low, and biomass loss is concentrated in a few large trees, but can also be associated with comparatively low AGB if stem mortality rates are high, and mortality is concentrated in a larger number of smaller trees (Fig. 4). Stem mortality rates may influence AGB because they affect the size structure of forests: demographic theory demonstrates how higher stem mortality rates are associated with a steeper slope of tree size/frequency distributions and therefore fewer large trees (Coomes *et al*., 2003; Muller‐Landau *et al*., 2006). In turn, variation in the number of large trees is a key predictor of spatial variation in biomass among forest plots (e.g. Baker *et al*., 2004; Baraloto *et al*., 2011). Importantly, this result indicates that incorporating stem diameter distributions within modelling frameworks will be important for obtaining accurate predictions of AGB.

Thirdly, our results resolve a paradox in the original paradigm – that *W*_P_ showed a negative correlation with AGB (Malhi, 2012). Here, with a broader range of sites, the expected positive correlation is found, although the strength of the relationship remains weak (Fig. 4a). Positive correlations between AGB and *W*_P_ are a feature of the output of DGVMs (e.g. Fig. 7). This analysis, at least to an extent, demonstrates consistency between one aspect of the models and the data, although the strength of the observed relationship is much weaker than that specified by the models (Figs 4a and 7).

Fourthly, the vertical offsets of the relationships between stem mortality rates and AGB among regions suggest that variation in the identity and height/diameter allometry of trees in different parts of Amazonia is also important for understanding variation in AGB. For example, observations from plots on the Guiana Shield show that these forests have very high AGB values for a given stem mortality rate (Fig. 4c), associated with surprisingly high *W*_P_ (Fig. 4a). This result implies that AGB is concentrated within trees with greater heights and/or higher wood density in these forests compared with other regions. A combination of good soil structural properties that promotes low stem mortality rates, and relatively high soil phosphorus concentrations that promote high productivity (Quesada *et al*., 2012) could conceivably allow these forests to attain the combination of high basal area, tree heights and wood density that results in particularly high AGB. Comparatively high levels of soil fertility are possible as this region may receive significant additions of inorganic phosphorus and other mineral nutrients from dust deposits; this region of the Amazon is believed to receive the highest amounts of dust from Saharan Africa (Mahowald *et al*., 1999, 2005). Alternatively, the greater heights, wood density and *W*_P_ of these forests may be related to their distinctive taxonomic composition; these forests contain a high proportion of stems of large‐statured species of Leguminosae (ter Steege *et al*., 2006). These species may achieve greater phosphorus‐use efficiency during photosynthesis or allocate a greater proportion of NPP to woody growth – both are processes that lead to higher AGB forests (Malhi, 2012). Variation in species composition, or biogeography, related to historical patterns of species dispersal over long timescales is known to be a factor in determining the high AGB and *W*_P_ of forests in Borneo compared with Amazonia (Banin *et al*., 2014). Similar processes may also be important within Amazon forests.

Conversely, forests on the Brazilian Shield towards the southern margins of Amazonian forests have particularly low AGB for a given stem mortality rate, associated with generally low values for *W*_P_ and high values of μ (Marimon *et al*., 2014; Fig. 4). Such low woody productivity, high stem mortality rates and potentially low stature forest in these locations are likely to be caused by repeated moisture stress and/or fire (Phillips *et al*., 2009; Brando *et al*., 2014): towards the southern margins of Amazonia, AGB approximately halves with a doubling in moisture stress quantified using the maximum climatological water deficit (Malhi *et al*., 2015).

Overall, our findings emphasize the pre‐eminent role of variation in stem mortality rates for controlling AGB, but indicate that variation in woody NPP is also important. They also emphasize how the links between AGB, tree growth and mortality are modified by species composition and the allocation of carbon to dense or light wood, or growth in height (Fig. 4c). Clearly, more comprehensive analyses of these sites including environmental data (cf Quesada *et al*., 2012) are required to tease apart the underlying drivers of these patterns. Additional data from low AGB forests in stressful environments across Amazonia, such as on white sand or peat (Baraloto *et al*., 2011; Draper *et al*., 2014), would also be valuable. Such low AGB forests have typically been excluded from ecosystem monitoring but may prove particularly informative to constrain the form of the relationships between *W*_P_, stem mortality rates and AGB.

Finally, our results suggest that the sensitivity of AGB to variation in stem mortality rates is greater in high AGB forests which have the lowest stem mortality rates (Fig. 4c). Increasing mortality rates are a feature of many threats faced by tropical forests, whether driven by increased growth, drought or fire, and extrapolations from forest plot data have been used to argue that such increases may substantially reduce the carbon stocks and carbon sink potential of these ecosystems (e.g. Lewis, 2006; Brienen *et al*., 2015). Our results indicate that forests with the highest AGB values will be most sensitive to a given increase in stem mortality rates (Fig. 4c). In addition, our results suggest that there may be regional differences in the sensitivity of the carbon stocks of Amazonian forests to changing stem mortality rates. For example, increases in stem mortality rates in the Guianas will not lead these forests to become structurally identical to western Amazon forests; they will follow their own trajectory related to their distinctive composition (Fig. 4c).

### Understanding spatial patterns in model simulations

Simulated AGB in the four DVGMs depends on the balance of woody NPP and losses due to the turnover of woody tissue, ‘background’ mortality, specific processes such as drought, or more generic ‘disturbance’ (Table 4). Here, we consider how these models simulate woody NPP and mortality to understand simulated patterns of AGB.

**Table 4:** Comparison of woody biomass mortality/turnover schemes used by the four DGVMs of this study. Where specific values are provided, these relate to the dominant PFT assumed by the models over our area of study

	INLAND	JULES	LPJmL	ORCHIDEE
1. Turnover of woody tissue				
Fixed/variable	Fixed	Fixed	Variable	Fixed
Woody turnover time (*τ*_w_)	25 years	200 years		30 years
2. Background disturbance rate				
Yes/No?	Yes	Yes	No	No
% a^−1^	0.05	0.05		
3. Specific drivers of mortality				
Negative carbon balance	No	No	Yes	No
Fire	Yes	No	Yes	No
Drought	No	No	Yes	No
Competition for light	No	Yes	Yes	No
References	Kucharik *et al*. (2000)	Clark *et al*. (2011)	Sitch *et al*. (2003)	Delbart *et al*. (2010)

DGVMs, dynamic global vegetation models.

**Figure 5:**
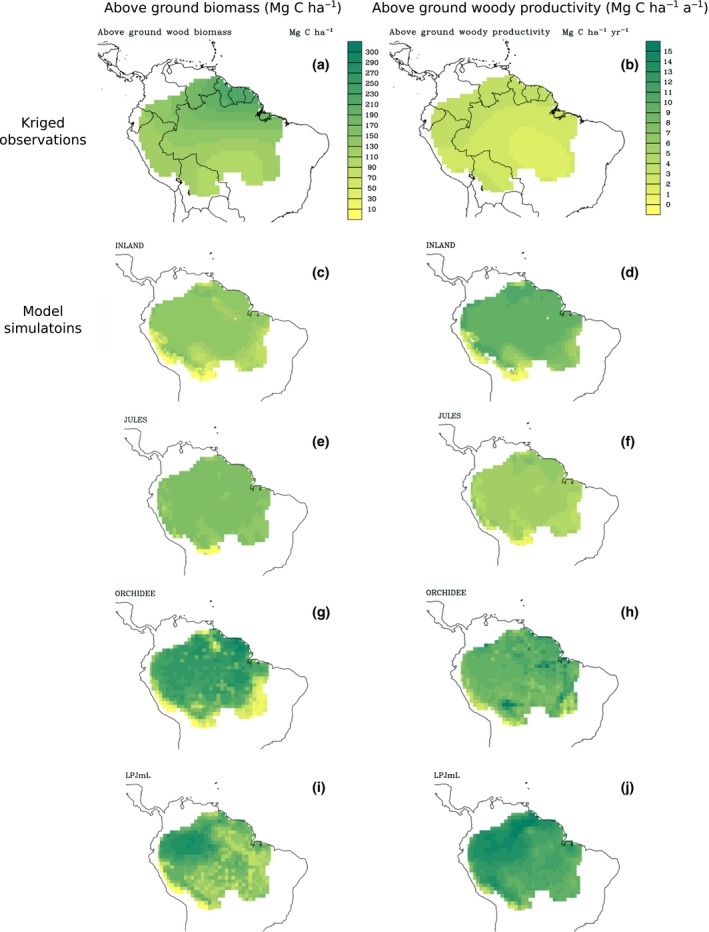
Kriged maps of above‐ground biomass and woody productivity from RAINFOR forest plot observations and simulated mean above‐ground biomass and woody NPP for 2000–2008 for four DGVMs. All maps are presented on the same scale; Fig. S7 displays kriged maps of the observations on independent scales. NPP, net primary productivity; DGVMs, dynamic global vegetation models.

**Figure 6:**
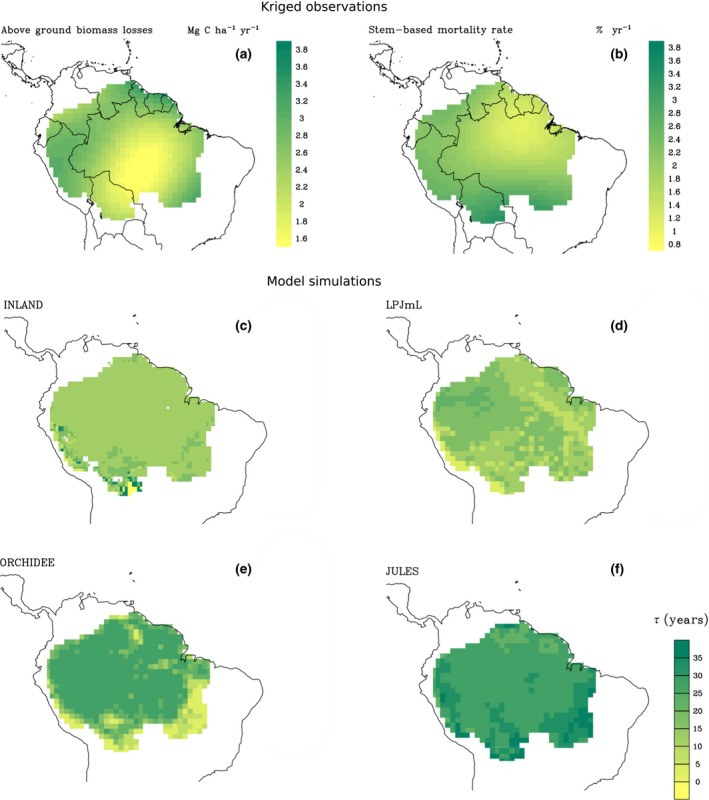
Kriged maps of (a) above‐ground biomass losses and (b) stem mortality rates from RAINFOR forest plot observations and simulated mean residence time (*τ* = AGB/*W*_P_) for 2000‐2008 for four DGVMs: (c) INLAND, (d) LPJmL, (e) ORCHIDEE and (f) JULES. DGVMs, dynamic global vegetation models; AGB, above‐ground biomass.

**Figure 7:**
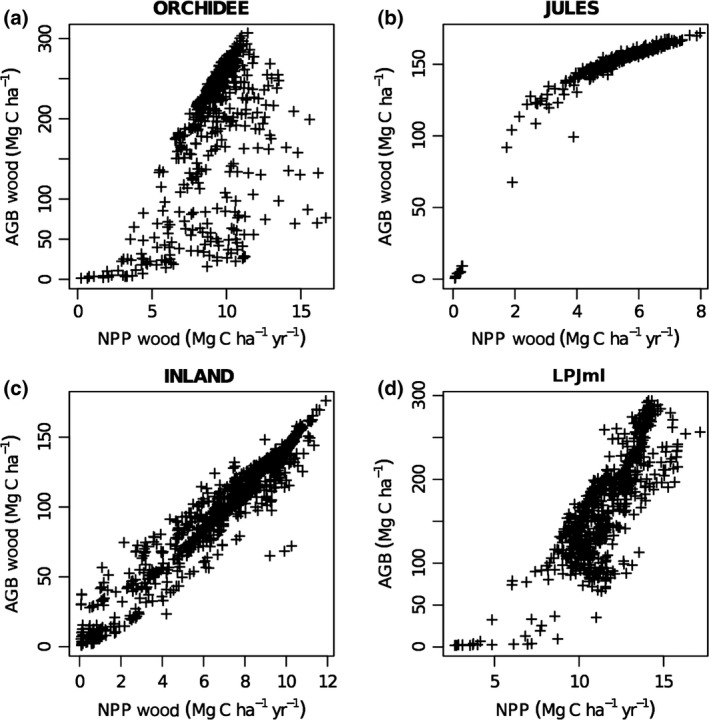
Simulated mean above‐ground wood biomass (2000‐2008) against simulated mean above‐ground woody net primary productivity (2000‐2008) for four DGVMs: (a) ORCHIDEE, (b) JULES, (c) INLAND and (d) LPJmL. DGVMs, dynamic global vegetation models.

Woody NPP in JULES is not responsive to the variability in climate and soils across the main part of the Amazon basin and this model therefore simulates little variation in *W*_P_ across this region (Fig. 5). This pattern translates into little variation in simulated AGB across much of Amazonia because mortality is essentially constant in JULES (Table 4) and simulated *τ*_w_ is largely invariant (Figs 5 and 6). As a result, there is a positive relationship between simulated AGB and NPP for this model (Fig. 7). However, interestingly, the relationship between AGB and NPP in JULES is nonlinear and suggests that there is an upper limit to the amount of AGB that can be simulated in JULES. This arises from the particular allocation scheme used in JULES where NPP is partitioned into biomass growth of existing vegetation or into ‘spreading’ of vegetated area (Cox, 2001). This partitioning into growth/spreading is regulated by LAI so that as LAI increases, less NPP is allocated to biomass growth. In this formulation, a maximum LAI value is prescribed which effectively sets a cap on biomass growth in the model, as at this point all of the NPP is directed into ‘spreading’ and none of it into growth of the existing vegetation. When a PFT occupies all of the available space in a grid cell and therefore cannot expand in area, all of the NPP effectively enters the litter via an assumed ‘self‐shading’ effect (Table 4; Huntingford *et al*., 2000).

INLAND simulates slightly more variation in *W*_P_ across the basin than JULES. However, most of this variation is observed at the basin fringes, which may be explained by INLAND's nonlinear relationship between *W*_P_ and rainfall; where annual rainfall exceeds 2 m yr^−1^, simulated *W*_P_ does not vary with changes in precipitation (Fig. S9). As a result, there is a very strong relationship between AGB and NPP (Fig. 7), and AGB varies little across Amazonia, similar to JULES (Fig. 5).

Productivity in LPJmL is much more strongly related to rainfall and MWD than either JULES or INLAND (Fig. S9), which is consistent with previous studies that have shown LPJ to be more sensitive to soil moisture stress than other models such as MOSES‐TRIFFID, the precursor model to JULES (Galbraith *et al*., 2010). As a result, we observe more spatial variation across the basin in *W*_P_. More generally, mortality is also more complex in this model and is a function of negative growth, heat stress and bioclimatic limits and includes disturbance from fire (Table 4; Sitch *et al*., 2003). As result, in contrast to the other models, there are correlations between *τ*_w_, rainfall and MWD in LPJmL (Fig. S9) resulting in substantial spatial variation in AGB and the highest AGB values in the wet, north‐west of the basin.

ORCHIDEE also demonstrates spatial variation in *W*_P_ which is nonlinearly correlated with rainfall (Fig. S9). Carbon residence times and AGB in ORCHIDEE are similarly, but more strongly, correlated with rainfall and MWD than *W*_P_, and as a result, there is a greater variability in the relationship between AGB and NPP for this model (Fig. 7) and greater spatial variation in AGB (Fig. 5).

### How can we improve simulations of spatial variation in DGVMs based on the observations?

A possible explanation for some of the disparities between the observations and model simulations may be the differences in how disturbance influences both datasets: the forest plots will experience the full range of disturbances that occur in natural forest, whilst the simulations are limited to reflecting only the effect of modelled processes. However, in broad terms, the degree and intensities of disturbance are likely to be comparable: amongst the DVGMs in this study, mortality is modelled based on a wide range of relevant processes – a background rate due to tree senescence, competition for light, drought and externally forced disturbance (Table 4). Rare but intense, large‐scale disturbances related to blowdowns are excluded from the simulations and such disturbances can have landscape‐scale effects (Chambers *et al*., 2013), but their extreme rarity and patchiness at a regional scale makes it unlikely that they substantially alter or determine broad scale patterns of forest structure and dynamics (Espírito‐Santo *et al*., 2014).

A key finding from the observational data is that variation in stem mortality rates determines spatial variation in AGB (Fig. 3). This finding implies that mortality must be modelled on the basis of individual stems, and suggests stem‐size distributions are important for predicting variation in AGB. However, the architecture of the DVGMs in this study does not incorporate stem‐size distributions, or individual‐based mortality rates. In contrast, three of the four models in this study employ a fixed value of *τ*_w_ (a PFT‐specific woody turnover rate, Table 4), to model a background rate of woody biomass loss, related to growth. In the models where these constant terms dominate mortality (e.g. JULES/INLAND), inevitably, the patterns of AGB mirror those of *W*_P_ and do not match the observations. Even in ORCHIDEE which simulates the highest biomass in the north‐east of the basin similar to the observations (Fig. 5), this apparent correspondence between the model and observations is not because this model effectively models tree mortality: like JULES and INLAND, ORCHIDEE also employs a constant mortality rate (Table 4; Delbart *et al*., 2010). In addition, the finding that variation in stem mortality determines variation in AGB implies that introducing simple relationships between mortality and *W*_P_, such as linking *τ*_w_ to NPP (Delbart *et al*., 2010) will not improve predictions for the whole basin. For example, the forests of the Guiana Shield, where forests have high *W*_P_ and high AGB but low stem mortality rates, will not be accurately modelled using the technique employed by Delbart *et al*. (2010).

A second key reason for discrepancies between the observations and models is that the key processes driving variation in the observations differ from the modelled processes. For example, when mortality is included as a dynamic process in the DGVMs, such as in LPJmL, mortality strongly reflects the variability in that process: moisture stress across the basin in the context of LPJmL. In contrast, stem mortality rates in Amazonian plots ultimately strongly respond also to edaphic properties such as soil physical properties (Quesada *et al*., 2012).

These findings suggest several ways in which vegetation models could be developed. Firstly, mortality needs to be effectively incorporated in these models, preferably through incorporating stem mortality rates (μ), rather than average carbon residence times (*τ*_w_), as a means of modelling the loss of woody carbon. The process of stem mortality is much more amenable for linking with the ultimate drivers of tree death, such as hydraulic failure, and is the key driver of variation in the size structure and AGB of Amazonian forests. We note that there have been positive advances in modelling mortality processes more mechanistically in DGVMs (e.g. Fisher *et al*., 2010, 2015) and that there is a considerable focus at present in improving the representation of vegetation dynamics in DGVMs (e.g. Verbeeck *et al*., 2011; De Weirdt *et al*., 2012; Castanho *et al*., 2013; Haverd *et al*., 2014; Weng *et al*., 2015). Secondly, DGVMs need to focus on including more functional diversity and variation in height/diameter relationships to capture regional differences in the carbon dynamics of Amazon forests. Thirdly, mortality processes need to be linked to edaphic properties such as a measure of soil structure/stability, and *W*_P_ to spatially varying soil nutrients to ensure that not only climate stress influences the spatial variation of AGB that is predicted by DGVMs. Finally, our study highlights the importance of size structure in shaping forest dynamics. To model tropical forest dynamics effectively, ‘average individual’ approaches which do not account for size distributions in tropical forests are insufficient. Several different aspects of these recommendations are already being implemented in emerging model frameworks (e.g. Fyllas *et al*., 2014; Sakschewski *et al*., 2015) and we look forward to testing the predictions of the next generation of vegetation models against baseline datasets of forest structure and dynamics.

## Supporting information


**Appendix S1**: Calculating above ground woody productivity (WP) from inventory data following Talbot *et al*. (2014).
**Appendix S2**: Description of the four DVGMs
