# Supplementary material for: Variation in stem mortality rates determines patterns of above‐ground biomass in Amazonian forests: implications for dynamic global vegetation models
Source: Glob Chang Biol. 2016 May 19;22(12):3996–4013. doi: 10.1111/gcb.13315 (PMC6849555; doi:10.1111/gcb.13315)
Supplement: Supplementary file 1 — Appendix S1: Calculating above ground woody productivity (WP) from inventory data following Talbot et al. (2014). Appendix S2: Description of the four DVGMs [file GCB-22-3996-s001.docx]

**Supplementary information**

**Appendix S1**

*Calculating above ground woody productivity (W_P_) from inventory data following Talbot et al. (2014)*

Above ground productivity was summed over four components: the growth of surviving trees during the census interval, the biomass of new recruits, the growth of trees that died during the census interval prior to their death, and the growth of unobserved recruits that also subsequently died during the monitoring period. We consider each component in turn.

Growth for each surviving tree with a diameter at breast height (dbh) ≥10 cm in both censuses was calculated as the difference in biomass estimates at each census using Equation 1 (Chave *et. al.,* 2005).

The total biomass of new recruits, i.e. trees which surpassed 10 cm dbh during the census interval, was used to estimate their contribution to woody productivity. Assuming that the forest is at equilibrium, this procedure effectively accounts for the growth of stems smaller than 10 cm diameter in the final estimate of stand-level, woody productivity (Talbot *et. al.* 2014).

For trees that were observed in the first census but died during the monitoring period, it was assumed that they died at the mid-point of the interval and thus grew for half of the interval length. The growth rate assigned to these trees was the median growth rate of the size class (10-19.9 cm; 20-39.9 cm or 40+ cm) of each dead tree.

Some trees are likely to have grown beyond 10 cm diameter during the census interval, and then died before they were recorded (Talbot *et al.* 2014). The number of such ‘unobserved recruits’ (*U*) was estimated as:

|  | *U* = *N* x *M* x *R* x *t* |  |
| --- | --- | --- |

where *N* equals the number of stems in the plot, *M* is the mean mortality rate, *R* is the mean recruitment rate and *t* is the census interval length. The contribution of *U* to total woody productivity was calculated by assigning these stems the median growth rate of trees in the 10-20 cm size class, and assuming that the recruits grow over only one-third of the census interval.

These four components were summed to give plot total productivity. For plots with multiple censuses, productivity was calculated for each census interval and an overall plot-level mean was calculated across all censuses, weighted by census interval length.

*References*

Chave J, Andalo C, Brown S *et al.* (2005) Tree allometry and improved estimation of carbon stocks and balance in tropical forests. *Oecologia,* **145**, 87-99.

Talbot J, Lewis SL, Lopez-Gonzalez G *et al.* (2014) Methods to estimate aboveground wood productivity from long-term forest inventory plots. *Forest Ecology and Management,* **320**, 30-38.

**Appendix S2**

*Description of the four DVGMs*

Four dynamic global vegetation models were used in this study: JULES (Best *et al.* 2011, Clarke *et al.* 2011), INLAND (Costa *et al.*, in prep), LPJml (Sitch *et al.* 2003, Gerten *et al.* 2004, Bondeau *et al.* 2007) and ORCHIDEE (Krinner *et al.* 2005). The Joint UK Land Environment Simulator (JULES) is the UK community land surface model (Best *et al.* 2011, Clark *et al.* 2011) and the land surface scheme for the Hadley Centre climate model. It is closely based on the MOSES-TRIFFID land surface scheme (Cox 2001), which was used in some of the first studies that predicted ‘die-back’ of the Amazon region. This study utilized version 2.1 of JULES. The Integrated Model of Land Surface Processes (INLAND) is the land surface module currently under development for the Brazilian Earth System Model, within the Brazilian scientific community. It is originally based on IBIS model (Foley *et al.*, 1996, Kucharik *et al.*, 2000), and further adapted with special focus on the representation of tropical ecosystems of South America.  The Lund-Potsdam-Jena Dynamic Global Vegetation Model for managed Land (LPJmL DGVM) is a process-based vegetation and hydrology model that builds upon the original LPJ model by including processes linked to land management. LPJ has been previously used in a number of studies investigating the dynamics of Amazonian rainforests under alternative climate regimes (e.g. Galbraith *et al.* 2010, Rammig *et al.* 2010, Poulter *et al.* 2010). The ORCHIDEE model consists of a DGVM coupled to the SECHIBA land-surface model (Ducoudré *et al.*, 1993). ORCHIDEE has been previously evaluated against data from flux tower sites (Verbeeck *et al.* 2011) and forest plot data (Delbart *et al.* 2010).

For each of the four DGVMs, we present brief descriptions of the key processes that affect biomass dynamics, including photosynthesis, carbon allocation and carbon turnover, as well as the treatment of plant functional types (PFTs). For full descriptions of the models, readers should refer to the original publications.

*JULES*

JULES simulates five PFTs: broadleaf trees, needleleaf trees, shrubs, C3 grasses and C4 grasses, which compete with each other following Lotka-Volterra dynamics (Cox 2001). Over Amazonia, broadleaf trees are the dominant plant functional type. In our simulations, a four-layer soil model is simulated with a total depth of 10 m, although individual plant functional types differ in their rooting depth. Net leaf photosynthesis is calculated based on Collatz *et al.* (1991, 1992). Leaf photosynthesis is coupled to stomatal conductance through the leaf internal CO_2_ concentration, calculated using the approach of Jacobs *et al.* (1994). Leaf photosynthesis is scaled to canopy level using a multi-layer approach which adopts the 2-stream approximation of radiation interception from Sellers *et al.* (1985). JULES simulates 3 vegetation pools (foliage, roots and wood), with maintenance respiration for each pool calculated dependent on tissue temperature and nitrogen content. Carbon fluxes from JULES are accumulated and passed to the TRIFFID vegetation dynamics model every 10 days. NPP is partitioned into a fraction used for growth of existing vegetation and a fraction for ‘spreading’ (Clark *et al.* 2011), based on the leaf area index. Tree mortality is not explicitly considered in the model. Biomass losses occur via turnover of carbon pools, each with specific turnover times, and prescribed large-scale disturbance rates.

*INLAND*

INLAND simulates 12 different PFTs competing for available resources within the grid cell and the relative success of each PFT determines its fractional coverage. The model allows trees and herbaceous plants or grasses to experience different light and water availability: while trees in the upper canopy have priority to capture available light (thus shading the shrubs and grasses in the lower part of the canopy), the herbaceous plants are able to capture soil water first when it infiltrates the ground (Foley *et al.*, 1996).

INLAND uses the mechanistic treatment of canopy photosynthesis proposed by Farquhar *et al.* (1980) and the semi-mechanistic Ball-Berry approach to estimate stomatal conductance (Ball *et al.* 1987; Collatz *et al.* 1991, 1992), computing gross photosynthesis, maintenance respiration and growth respiration to yield the annual carbon balance for each PFT. The vegetation dynamics module simulates biomass changes for each PFT on a yearly time step. Net primary productivity (NPP) is allocated to individual biomass pools (leaves, roots, wood) according to fixed allocation coefficients. Mortality is not explicitly modelled. Instead, biomass losses occur via turnover of the existing carbon pool, according to fixed turnover rates as well as via large-scale disturbance caused by fire or land use change.

*LPJmL*

In LPJml, most physiological and hydrological processes are simulated at daily time steps, whereas vegetation dynamics and PFT composition are updated annually. Natural vegetation is represented by nine plant functional types (PFTs) which describe the main characteristics of plants within the different biomes across the globe. Over Amazonia, the dominant PFTs are tropical evergreen trees and tropical raingreen trees. Photosynthesis is based on the Farquhar model approach (Farquhar *et al.*, 1980; Farquhar and Von Caemmerer, 1982) with air temperature and radiation controlling photosynthetic activity at the leaf level. Transpiration and photosynthesis are coupled through stomatal conductance of the leaves, where increasing transpirational losses or carbon starvation due to closed stomata can reduce NPP under drought conditions or high temperatures. With continued drought depleting soil water storage, tropical raingreen trees shed their leaves during the dry season to avoid carbon loss and mortality. Tropical evergreen broadleaf trees keep their leaves and are thus usually outcompeted in a seasonal dry tropical climate.

Carbon gained is allocated annually to the living carbon pools where basic allometric relations between crown area, tree height and stem diameter are met (Sitch *et al.* 2003). The pipe model ensures that each unit of leaf area is supported by a corresponding area of transport tissue, i.e. the sapwood cross-sectional area. Canopy closure is assumed but no crown overlap is permitted. Furthermore, plants can invest more carbon to fine roots under water-limited conditions to reduce drought risks This term is parameterized for each PFT.

Tree mortality results from heat stress, fire and light competition. The latter can occur due to low growth efficiency or thinning effects. Mortality from heat stress occurs when a PFT-specific temperature is crossed (Sitch *et al.* 2003), and individuals lost through fire are quantified by a PFT-specific parameter describing fire intensity and severity (Thonicke *et al.* 2001).

*ORCHIDEE*

Photosynthesis in ORCHIDEE is simulated following the formulations of Farquhar *et al.* (1980) and Collatz *et al.* (1992), while stomatal conductance is computed via the technique of Ball *et al.* (1987). Maintenance respiration of plant pools in ORCHIDEE is calculated using PFT-specific functions of (a) temperature and biomass and (b) nitrogen/carbon ratios (see Ruimy *et al.*, 1996). Soil layering characteristics are site dependent, with rooting distributions determined by availability of water, light and nitrogen. By definition, vegetation phenology is prognostic and is based on PFT-specific temperature and moisture constraints (Krinner *et al.*, 2005). With respect to biomass pools, the model consists of four separate carbon pools, plus total soil carbon (Verbeeck *et al.*, 2011). Representation of vegetation dynamics and disturbance follows the approach described in the LPJ model (Sitch *et al.*, 2003). For the simulations in this study an 11 layer soil hydrology scheme was used (Guimberteau *et al.* 2012).

*References*

Ball, JT, Woodrow IE, and Berry JA (1987) A model predicting stomatal conductance and its contribution to the control of photosynthesis under different environmental conditions. In *Progress in Photosynthesis Research*, (ed Biggens J), pp 221-224, Springer, The Netherlands.

Best M, Pryor M, Clark D *et al.* (2011) The Joint UK Land Environment Simulator (JULES), model description–Part 1: energy and water fluxes. *Geoscientific Model Development,* **4**, 677-699.

Bondeau A, Smith PC, Zaehle S *et al.* (2007) Modelling the role of agriculture for the 20th century global terrestrial carbon balance. *Global Change Biology,* **13**, 679-706.

Clark D, Mercado L, Sitch S *et al.* (2011) The Joint UK Land Environment Simulator (JULES), model description–Part 2: carbon fluxes and vegetation dynamics. *Geoscientific Model Development,* **4**, 701-722.

Collatz GJ, Ball JT, Grivet C, Berry JA (1991) Physiological and environmental regulation of stomatal conductance, photosynthesis and transpiration: a model that includes a laminar boundary layer. *Agricultural and Forest Meteorology*, **54,** 107-136.

Collatz GJ, Ribas-Carbo M, Berry JA (1992) Coupled photosynthesis-stomatal conductance model for leaves of C4 plants. *Functional Plant Biology*, **19**, 519-538.

Cox PM (2001) Description of the TRIFFID dynamic global vegetation model. pp Page, Technical Note 24, Hadley Centre, United Kingdom Meteorological Office, Bracknell, UK.

Da Costa ACL, Galbraith D, Almeida S *et al.* (2010) Effect of 7 yr of experimental drought on vegetation dynamics and biomass storage of an eastern Amazonian rainforest. *New Phytologist,* **187**, 579-591.

Ducoudré NI, Laval K, Perrier A (1993) SECHIBA, a new set of parameterizations of the hydrologic exchanges at the land-atmosphere interface within the LMD atmospheric general circulation model. *Journal of Climate,* **6**, 248-273.

Delbart N, Ciais P, Chave J, Viovy N, Malhi Y, Le Toan T (2010) Mortality as a key driver of the spatial distribution of aboveground biomass in Amazonian forest: results from a dynamics vegetation model. *Biogeosciences,* **7**, 3017-3039.

Farquhar GD, von Caemmerer S, Berry JA (1980) A biochemical model of photosynthetic CO2 assimilation in leaves of C3 species. *Planta* **149,** 78-90.

Farquhar GD, von Caemmerer S (1982) Modelling of photosynthetic response to environmental conditions. *Physiological plant ecology II*. pp 549-587, Springer, Berlin Heidelberg

Foley JA, Prentice IC, Ramankutty N *et al.* (1996) An integrated biosphere model of land surface processes, terrestrial carbon balance, and vegetation dynamics. *Global Biogeochemical Cycles,* **10**, 603-628.

Galbraith D, Levy PE, Sitch S, Huntingford C, Cox P, Williams M, Meir P (2010) Multiple mechanisms of Amazonian forest biomass losses in three dynamic global vegetation models under climate change. *New Phytologist,* **187**, 647-665.

Gerten D, Schaphoff S, Haberlandt U, Lucht W, Sitch S (2004) Terrestrial vegetation and water balance—hydrological evaluation of a dynamic global vegetation model. *Journal of Hydrology,* **286**, 249-270.

Guimberteau M, Drapeau G, Ronchail J, *et al.* (2012). Discharge simulation in the sub-basins of the Amazon using ORCHIDEE forced by new datasets. *Hydrology and Earth System Sciences*, **16**, 911-935.

Jacobs C (1994) Direct impact of atmospheric CO_2_ enrichment on regional

transpiration, Ph.D. thesis, Wageningen Agricultural University.

Krinner G, Viovy N, De Noblet‐Ducoudré N *et al.* (2005) A dynamic global vegetation model for studies of the coupled atmosphere‐biosphere system. *Global Biogeochemical Cycles,* **19**.

Kucharik CJ, Foley JA, Delire C *et al.* (2000) Testing the performance of a dynamic global ecosystem model: water balance, carbon balance, and vegetation structure. *Global Biogeochemical Cycles*, **14**, 795-825.

Poulter *et al.* 2010. Net biome production of the Amazon Basin in the 21^st^ Century. *Global Change Biology* **16**, 2062-2075.

Rammig A, Jupp T, Thonicke K *et al.* (2010) Estimating the risk of Amazonian forest dieback. *New Phytologist,* **187**, 694-706.

Ruimy A, Dedieu G, Saugier B (1996) TURC: A diagnostic model of continental gross primary productivity and net primary productivity. *Global Biogeochemical Cycles* **10**, 269-285.

Sellers PJ (1985) Canopy reflectance, photosynthesis and transpiration. *International Journal of Remote Sensing* **6**, 1335-1372.

Sheffield J, Goteti G, Wood EF (2006) Development of a 50-year high-resolution global dataset of meteorological forcings for land surface modeling. *Journal of Climate,* **19**, 3088-3111.

Sitch S, Smith B, Prentice IC *et al.* (2003) Evaluation of ecosystem dynamics, plant geography and terrestrial carbon cycling in the LPJ dynamic global vegetation model. *Global Change Biology,* **9**, 161-185.

Thonicke K, Venevsky S, Sitch S, Cramer, W (2001). The role of fire disturbance for global vegetation dynamics: coupling fire into a Dynamic Global Vegetation Model. *Global Ecology and Biogeography*, ***10***, 661-677.

Verbeeck H, Peylin P, Bacour C, Bonal D, Steppe K, Ciais P (2011) Seasonal patterns of CO2 fluxes in Amazon forests: Fusion of eddy covariance data and the ORCHIDEE model. *Journal of Geophysical Research: Biogeosciences (2005–2012),* **116**.

Table S1. Aboveground biomass (AGB), woody productivity (*W_P_*) and woody biomass losses (*W_L_*) for 167 forest plots across Amazonia. AGB data is the same as in Mitchard *et al.* (2014). For the data on forest dynamics, the mean date of the first census is 2000.2 and the mean date of the final census is 2008.5; mean census interval length is 3.70 years and plot mean total monitoring period is 8.3 years. Regions (see also Fig. 1) are Western Amazonia (W), the Brazilian Shield (BrSh), East Central Amazonia (EC) and the Guiana Shield (GuSh).

| Plot code | Region | Latitude | Longitude | AGB | *W_P_* | *W_L_* | *μ* |
| --- | --- | --- | --- | --- | --- | --- | --- |
|  |  | deg. | deg. | Mg ha^-1^ | Mg C ha^-1^ a^-1^ | Mg C ha^-1^ a^-1^ | % yr^-1^ |
| AGJ-01 | W | -11.9 | -71.3 | 136.08 | 2.06 | 1.90 | 2.21 |
| AGP-01 | W | -3.7 | -70.3 | 127.04 | 3.55 | 2.70 | 1.28 |
| AGP-02 | W | -3.7 | -70.3 | 128.79 | 4.34 | 6.16 | 4.75 |
| ALF-01 | BrSh | -9.6 | -55.9 | 97.25 | 1.79 | 0.96 | 1.29 |
| ALM-01 | W | -11.8 | -71.5 | 125.60 | 2.97 | 2.91 | 1.82 |
| ALP-10 | W | -3.9 | -73.4 | 142.94 | 3.42 | 2.95 | 2.89 |
| ALP-11 | W | -3.9 | -73.4 | 130.51 | 3.42 | 2.95 | 2.89 |
| ALP-20 | W | -3.9 | -73.4 | 115.66 | 3.38 | 3.75 | 2.20 |
| ALP-21 | W | -3.9 | -73.4 | 130.82 | 3.38 | 3.75 | 2.20 |
| ALP-30 | W | -3.9 | -73.4 | 111.16 | 2.52 | 2.90 | 1.57 |
| ALP-40 | W | -3.9 | -73.4 | 106.32 | 3.74 | 1.06 | 1.47 |
| BAC-01 | W | 7.5 | -71.0 | 107.45 | 3.27 | 1.91 | 3.24 |
| BAC-02 | W | 7.5 | -71.0 | 94.96 | 2.39 | 0.51 | 3.92 |
| BAC-03 | W | 7.5 | -71.0 | 197.31 | 2.90 | 0.63 | 2.49 |
| BAC-04 | W | 7.5 | -71.0 | 151.18 | 2.61 | 0.63 | 2.29 |
| BAC-05 | W | 7.5 | -71.0 | 57.09 | 2.25 | 1.97 | 4.26 |
| BAC-06 | W | 7.5 | -71.0 | 129.16 | 3.24 | 3.10 | 2.58 |
| BAR-01 | W | -11.9 | -71.4 | 167.28 | 4.00 | 1.80 | 1.51 |
| BDF-01 | EC | -2.3 | -60.1 | 172.58 | 2.27 | 1.84 | 1.09 |
| BDF-03 | EC | -2.4 | -59.8 | 160.11 | 3.00 | 2.74 | 1.47 |
| BDF-04 | EC | -2.4 | -59.8 | 148.84 | 2.45 | 2.45 | 2.15 |
| BDF-05 | EC | -2.4 | -59.8 | 133.94 | 1.86 | 1.28 | 1.40 |
| BDF-06 | EC | -2.4 | -59.9 | 141.88 | 2.97 | 2.43 | 1.58 |
| BDF-07 | EC | -2.4 | -59.9 | 163.66 | 2.45 | 1.71 | 1.19 |
| BDF-08 | EC | -2.4 | -59.9 | 157.74 | 2.07 | 2.33 | 1.53 |
| BDF-09 | EC | -2.4 | -59.9 | 179.65 | 2.53 | 1.92 | 1.22 |
| BDF-10 | EC | -2.4 | -59.9 | 147.97 | 2.04 | 1.68 | 1.25 |
| BDF-11 | EC | -2.4 | -59.9 | 174.16 | 1.93 | 1.51 | 1.11 |
| BDF-12 | EC | -2.4 | -59.9 | 202.89 | 2.24 | 3.58 | 1.53 |
| BDF-13 | EC | -2.4 | -59.9 | 166.76 | 2.40 | 2.13 | 1.18 |
| BDF-14 | EC | -2.4 | -60.0 | 173.06 | 1.99 | 1.77 | 0.74 |
| BEE-01 | W | -16.5 | -64.6 | 113.01 | 3.13 | 1.33 | 1.83 |
| BEE-05 | W | -16.5 | -64.6 | 102.00 | 3.33 | 1.57 | 1.74 |
| Plot code | Region | Latitude | Longitude | AGB | *W_p_* | *W_l_* | *μ* |
|  |  | deg. | deg. | Mg ha^-1^ | Mg C ha^-1^ a^-1^ | Mg C ha^-1^ a^-1^ | % yr^-1^ |
| BNT-01 | EC | -2.6 | -60.2 | 169.78 | 2.23 | 1.74 | 1.02 |
| BNT-02 | EC | -2.6 | -60.2 | 164.64 | 2.21 | 1.62 | 0.78 |
| BNT-04 | EC | -2.6 | -60.2 | 146.73 | 2.40 | 1.29 | 1.11 |
| BOG-01 | W | -0.7 | -76.5 | 136.12 | 4.30 | 3.23 | 2.30 |
| BOG-02 | W | -0.7 | -76.5 | 99.16 | 3.54 | 2.97 | 2.63 |
| CAI-04 | W | 8.7 | -70.1 | 121.08 | 4.17 | 2.20 | 5.64 |
| CAX-01 | EC | -1.7 | -51.5 | 184.55 | 2.67 | 1.20 | 0.79 |
| CAX-02 | EC | -1.7 | -51.5 | 181.91 | 2.56 | 3.14 | 1.38 |
| CAX-06 | EC | -1.7 | -51.5 | 205.79 | 2.72 | 4.43 | 1.36 |
| CAX-08 | EC | -1.8 | -51.5 | 117.01 | 3.44 | 1.20 | 2.05 |
| CHO-01 | BrSh | -14.4 | -61.2 | 54.82 | 1.85 | 1.81 | 5.37 |
| CPP-01 | BrSh | -1.8 | -47.1 | 161.18 | 2.50 | 1.77 | 1.72 |
| CRP-01 | BrSh | -14.5 | -61.5 | 76.00 | 1.68 | 1.48 | 2.65 |
| CRP-02 | BrSh | -14.5 | -61.5 | 90.99 | 2.59 | 2.15 | 2.67 |
| CUZ-01 | W | -12.5 | -69.1 | 126.43 | 2.87 | 4.16 | 5.33 |
| CUZ-02 | W | -12.5 | -69.1 | 116.63 | 2.85 | 2.25 | 2.30 |
| CUZ-03 | W | -12.5 | -69.1 | 107.97 | 3.06 | 1.94 | 2.06 |
| CUZ-04 | W | -12.5 | -69.1 | 130.38 | 3.80 | 3.92 | 3.13 |
| DOI-01 | W | -10.6 | -68.3 | 114.99 | 2.46 | 1.67 | 1.95 |
| DOI-02 | W | -10.5 | -68.3 | 73.76 | 2.51 | 2.12 | 5.59 |
| ECE-01 | W | 10.7 | -75.3 | 62.51 | 1.77 | 0.81 | 0.68 |
| ELD-01 | GuSh | 6.1 | -61.4 | 216.26 | 5.25 | 3.45 | 1.67 |
| ELD-02 | GuSh | 6.1 | -61.4 | 258.82 | 3.46 | 0.22 | 0.43 |
| ELD-03 | GuSh | 6.1 | -61.4 | 136.48 | 4.30 | 8.48 | 2.99 |
| ELD-04 | GuSh | 6.1 | -61.3 | 145.73 | 3.79 | 2.58 | 1.41 |
| FMH-01 | GuSh | 5.2 | -58.7 | 373.44 | 3.91 | 0.42 | 0.10 |
| GMT-01 | EC | -1.1 | -47.8 | 159.82 | 3.52 | 3.10 | 1.58 |
| IWO-21 | GuSh | 4.6 | -58.7 | 188.88 | 2.51 | 1.20 | 0.79 |
| IWO-22 | GuSh | 4.6 | -58.7 | 296.91 | 2.35 | 0.77 | 0.50 |
| JAC-01 | EC | -2.6 | -60.2 | 145.09 | 2.22 | 2.08 | 1.20 |
| JAC-02 | EC | -2.6 | -60.2 | 142.69 | 2.19 | 2.15 | 1.34 |
| JAS-02 | W | -1.1 | -77.6 | 112.42 | 3.56 | 5.41 | 3.56 |
| JAS-03 | W | -1.1 | -77.6 | 115.79 | 3.55 | 1.57 | 1.48 |
| JAS-04 | W | -1.1 | -77.6 | 140.56 | 4.51 | 1.83 | 1.39 |
| JEN-11 | W | -4.9 | -73.6 | 135.42 | 2.85 | 3.40 | 2.40 |
| JEN-12 | W | -4.9 | -73.6 | 117.78 | 2.08 | 1.17 | 1.06 |
| LAS-02 | W | -12.6 | -70.1 | 122.63 | 3.09 | 2.62 | 2.51 |
| LCA-13 | BrSh | -15.7 | -62.8 | 81.28 | 2.31 | 1.38 | 2.55 |
| LCA-16 | BrSh | -15.7 | -62.8 | 52.06 | 2.42 | 1.53 | 3.30 |
| LFB-01 | BrSh | -14.6 | -60.8 | 113.98 | 2.72 | 1.38 | 3.26 |
| LFB-02 | BrSh | -14.6 | -60.8 | 136.27 | 2.97 | 2.20 | 3.06 |
| LOR-01 | W | -3.1 | -70.0 | 136.95 | 4.60 | 8.22 | 2.45 |
| LOR-02 | W | -3.1 | -70.0 | 143.84 | 3.57 | 1.37 | 2.53 |
| LSL-01 | BrSh | -14.4 | -61.1 | 68.22 | 2.46 | 1.67 | 3.55 |
| LSL-02 | BrSh | -14.4 | -61.1 | 76.27 | 3.15 | 0.96 | 1.83 |
| MBT-01 | W | -10.1 | -65.9 | 102.75 | 2.13 | 1.00 | 1.62 |
| Plot code | Region | Latitude | Longitude | AGB | *W_p_* | *W_l_* | *μ* |
|  |  | deg. | deg. | Mg ha^-1^ | Mg C ha^-1^ a^-1^ | Mg C ha^-1^ a^-1^ | % yr^-1^ |
| MBT-04 | W | -10.3 | -65.5 | 115.98 | 2.65 | 1.33 | 1.18 |
| MBT-05 | W | -10.0 | -65.6 | 140.96 | 3.36 | 0.68 | 1.04 |
| MBT-06 | W | -10.0 | -65.6 | 115.24 | 3.04 | 1.00 | 1.40 |
| MBT-08 | W | -9.9 | -65.8 | 90.75 | 1.49 | 1.13 | 1.76 |
| MIN-01 | W | -8.6 | -72.9 | 105.40 | 2.94 | 2.23 | 2.27 |
| MNU-01 | W | -11.9 | -71.4 | 148.52 | 3.38 | 2.37 | 2.27 |
| MNU-03 | W | -11.9 | -71.4 | 112.07 | 3.43 | 3.53 | 3.28 |
| MNU-04 | W | -11.9 | -71.4 | 92.57 | 1.17 | 1.05 | 2.10 |
| MNU-05 | W | -11.9 | -71.4 | 172.18 | 2.90 | 4.21 | 2.49 |
| MNU-06 | W | -11.9 | -71.4 | 139.33 | 2.94 | 3.90 | 2.19 |
| MNU-09 | W | -12.0 | -71.2 | 148.51 | 3.19 | 3.02 | 2.47 |
| MTH-01 | W | -8.9 | -72.8 | 78.43 | 2.62 | 1.95 | 3.25 |
| NOU-01 | GuSh | 4.1 | -52.7 | 215.34 | 5.30 | 2.38 | 1.41 |
| NOU-02 | GuSh | 4.1 | -52.7 | 220.03 | 4.35 | 1.95 | 1.03 |
| NOU-03 | GuSh | 4.1 | -52.7 | 295.20 | 4.78 | 3.94 | 1.35 |
| NOU-04 | GuSh | 4.1 | -52.7 | 196.49 | 4.84 | 2.53 | 1.77 |
| NOU-05 | GuSh | 4.1 | -52.7 | 158.21 | 3.70 | 4.77 | 1.84 |
| NOU-06 | GuSh | 4.1 | -52.7 | 151.88 | 4.13 | 2.56 | 2.05 |
| NOU-07 | GuSh | 4.1 | -52.7 | 141.21 | 3.47 | 2.35 | 1.56 |
| NOU-08 | GuSh | 4.1 | -52.7 | 165.06 | 3.85 | 2.57 | 1.68 |
| NOU-09 | GuSh | 4.1 | -52.7 | 132.01 | 3.79 | 3.70 | 1.42 |
| NOU-10 | GuSh | 4.1 | -52.7 | 145.13 | 3.39 | 3.36 | 1.79 |
| NOU-11 | GuSh | 4.1 | -52.7 | 252.92 | 4.17 | 1.63 | 1.22 |
| NOU-12 | GuSh | 4.1 | -52.7 | 205.57 | 3.60 | 2.26 | 1.36 |
| NOU-13 | GuSh | 4.1 | -52.7 | 217.41 | 3.18 | 1.80 | 1.05 |
| NOU-14 | GuSh | 4.1 | -52.7 | 208.95 | 2.79 | 4.44 | 1.60 |
| NOU-15 | GuSh | 4.1 | -52.7 | 200.73 | 2.57 | 2.35 | 1.36 |
| NOU-16 | GuSh | 4.1 | -52.7 | 152.96 | 3.04 | 3.86 | 1.84 |
| NOU-17 | GuSh | 4.1 | -52.7 | 231.81 | 4.06 | 1.03 | 0.77 |
| NOU-18 | GuSh | 4.1 | -52.7 | 220.91 | 3.29 | 0.81 | 0.45 |
| NOU-19 | GuSh | 4.1 | -52.7 | 203.15 | 3.24 | 3.05 | 0.83 |
| NOU-20 | GuSh | 4.1 | -52.7 | 225.89 | 4.23 | 1.16 | 0.60 |
| NOU-21 | GuSh | 4.1 | -52.7 | 203.26 | 3.13 | 0.78 | 0.60 |
| NOU-22 | GuSh | 4.1 | -52.7 | 231.78 | 3.08 | 3.99 | 1.43 |
| PAR-20 | GuSh | 5.3 | -52.9 | 239.93 | 2.22 | 16.69 | 4.94 |
| PAR-21 | GuSh | 5.3 | -52.9 | 236.50 | 2.16 | 3.45 | 1.37 |
| PAR-22 | GuSh | 5.3 | -52.9 | 149.31 | 3.32 | 3.90 | 3.28 |
| PAR-23 | GuSh | 5.3 | -52.9 | 189.84 | 3.11 | 0.99 | 0.85 |
| PAR-24 | GuSh | 5.3 | -52.9 | 215.02 | 4.20 | 7.43 | 4.36 |
| PAR-26 | GuSh | 5.3 | -52.9 | 192.30 | 2.48 | 4.25 | 2.62 |
| PAR-27 | GuSh | 5.3 | -52.9 | 181.93 | 2.29 | 3.42 | 1.16 |
| PAR-28 | GuSh | 5.3 | -52.9 | 228.39 | 2.50 | 1.13 | 0.86 |
| PAR-29 | GuSh | 5.3 | -52.9 | 257.82 | 2.95 | 0.72 | 0.63 |
| PNY-04 | W | -10.3 | -75.2 | 107.40 | 2.80 | 4.04 | 4.66 |
| POR-01 | W | -10.8 | -68.8 | 152.27 | 3.13 | 2.06 | 2.31 |
| POR-02 | W | -10.8 | -68.8 | 103.36 | 2.72 | 2.21 | 2.72 |
| Plot code | Region | Latitude | Longitude | AGB | *W_p_* | *W_l_* | *μ* |
|  |  | deg. | deg. | Mg ha^-1^ | Mg C ha^-1^ a^-1^ | Mg C ha^-1^ a^-1^ | % yr^-1^ |
| PTB-01 | EC | -1.2 | -56.4 | 213.27 | 2.16 | 4.38 | 1.76 |
| PTB-02 | EC | -1.5 | -56.4 | 115.79 | 3.21 | 1.70 | 2.11 |
| RET-05 | W | -11.0 | -65.7 | 119.59 | 2.80 | 2.20 | 2.11 |
| RET-06 | W | -11.0 | -65.7 | 141.46 | 2.67 | 1.92 | 2.51 |
| RET-08 | W | -11.0 | -65.7 | 134.75 | 2.36 | 1.27 | 2.17 |
| RET-09 | W | -11.0 | -65.7 | 136.95 | 2.49 | 1.20 | 1.89 |
| RFH-01 | W | -9.8 | -67.7 | 137.67 | 2.43 | 6.01 | 2.80 |
| RIO-01 | GuSh | 8.1 | -61.7 | 222.48 | 3.92 | 4.55 | 1.52 |
| RIO-02 | GuSh | 8.1 | -61.7 | 222.09 | 3.99 | 2.87 | 1.90 |
| RST-01 | W | -9.0 | -72.3 | 108.49 | 3.19 | 1.96 | 1.66 |
| SCR-05 | GuSh | 1.9 | -67.0 | 191.71 | 3.16 | 1.87 | 0.74 |
| SCT-01 | W | -17.1 | -64.8 | 98.55 | 2.86 | 2.42 | 2.75 |
| SCT-06 | W | -17.1 | -64.8 | 94.54 | 3.53 | 3.28 | 4.41 |
| SUC-01 | W | -3.2 | -72.9 | 133.45 | 3.45 | 3.19 | 1.98 |
| SUC-02 | W | -3.2 | -72.9 | 135.35 | 3.16 | 3.38 | 2.22 |
| SUC-03 | W | -3.2 | -72.9 | 144.43 | 2.66 | 2.12 | 2.45 |
| SUC-04 | W | -3.2 | -72.9 | 141.05 | 3.19 | 2.32 | 1.59 |
| SUC-05 | W | -3.3 | -72.9 | 134.98 | 3.45 | 1.78 | 1.73 |
| TAM-01 | W | -12.8 | -69.3 | 114.18 | 2.60 | 1.48 | 1.61 |
| TAM-02 | W | -12.8 | -69.3 | 123.42 | 2.61 | 2.03 | 1.83 |
| TAM-03 | W | -12.8 | -69.3 | 144.54 | 2.55 | 0.70 | 0.86 |
| TAM-04 | W | -12.8 | -69.3 | 134.34 | 3.94 | 2.48 | 2.18 |
| TAM-05 | W | -12.8 | -69.3 | 118.71 | 3.36 | 1.92 | 2.34 |
| TAM-06 | W | -12.8 | -69.3 | 126.47 | 3.17 | 1.38 | 1.46 |
| TAM-07 | W | -12.8 | -69.3 | 128.15 | 3.03 | 4.23 | 2.69 |
| TAM-08 | W | -12.8 | -69.3 | 108.49 | 2.96 | 2.26 | 2.22 |
| TEC-01 | EC | -1.7 | -51.5 | 194.01 | 2.45 | 3.58 | 1.48 |
| TEC-02 | EC | -1.7 | -51.5 | 230.64 | 2.69 | 1.25 | 0.45 |
| TEC-03 | EC | -1.7 | -51.5 | 250.72 | 1.83 | 1.50 | 0.90 |
| TEC-04 | EC | -1.7 | -51.5 | 202.24 | 2.51 | 2.85 | 1.22 |
| TEC-05 | EC | -1.8 | -51.6 | 232.97 | 2.49 | 0.61 | 0.78 |
| TEC-06 | EC | -1.7 | -51.4 | 165.97 | 2.21 | 2.79 | 1.57 |
| TEM-01 | EC | -3.0 | -59.9 | 182.50 | 2.42 | 3.11 | 1.35 |
| TEM-02 | EC | -3.0 | -59.9 | 165.75 | 2.01 | 0.74 | 0.50 |
| TEM-03 | EC | -2.4 | -59.9 | 136.72 | 1.79 | 0.42 | 0.58 |
| TEM-04 | EC | -2.4 | -59.8 | 121.81 | 2.36 | 0.82 | 1.03 |
| TEM-05 | EC | -2.6 | -60.2 | 132.14 | 2.66 | 3.57 | 2.07 |
| TEM-06 | EC | -2.6 | -60.1 | 125.80 | 1.88 | 1.84 | 1.34 |
| TIP-02 | W | -0.6 | -76.1 | 94.25 | 2.65 | 1.79 | 1.70 |
| TIP-03 | W | -0.6 | -76.1 | 124.12 | 3.78 | 2.02 | 2.55 |
| YAN-01 | W | -3.4 | -72.8 | 132.79 | 3.68 | 3.32 | 2.45 |
| YAN-02 | W | -3.4 | -72.8 | 138.46 | 3.69 | 1.87 | 1.30 |

Figure S1. Cumulative number of plots monitored during 2000-8, and census periods covered by all plots used in this study. Period of model simulations also shown.


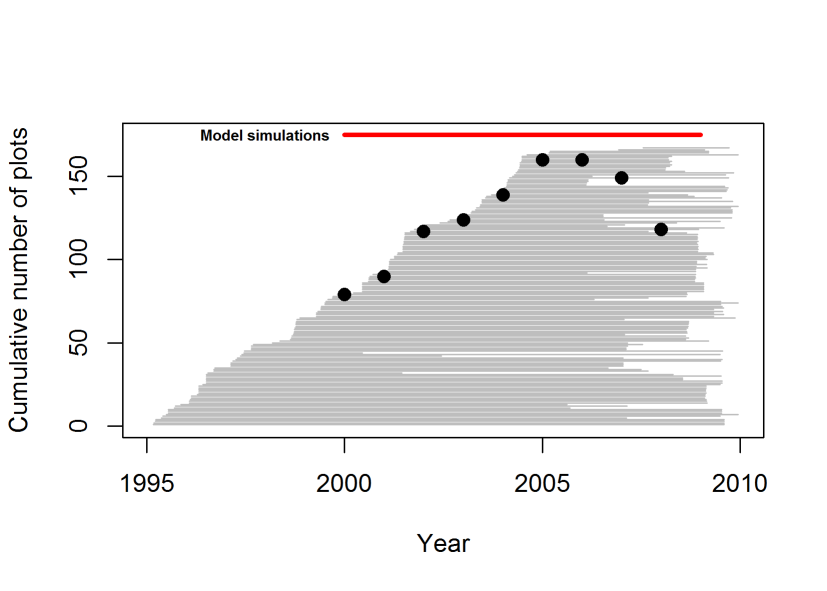


Figure S2. Semivariograms of variability in above ground biomass, above ground woody productivity and stem-based mortality rates with distance (km) based on RAINFOR long-term plot data from across Amazonia.


Figure S3. Mean precipitation, maximum cumulative water deficit (MWD), temperature and short wave radiation for 2000-2008 from the Sheffield *et al.*, (2006) meteorological forcings used in all model simulations.

Figure S4. Predicted values of above ground biomass, above ground woody productivity and stem-based mortality rates using a leave one out cross validation kriging method, versus the observed values.

Figure S5. Distribution of leave-one-out cross validation residuals across the RAINFOR plot network

Figure S6. Aboveground woody biomass (Mg C ha^-1^) as a function of stem number (≥10 cm diameter) for 167 forest plots across Amazonia


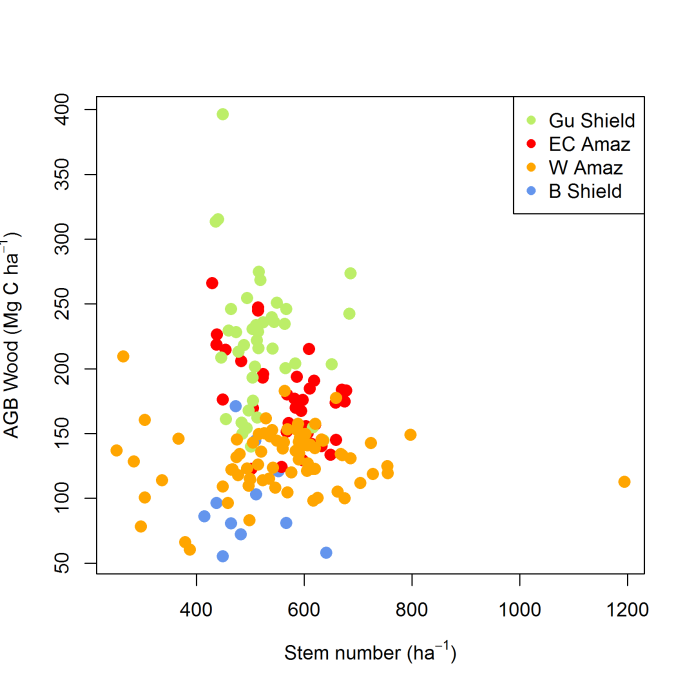


Figure S7. Kriged maps of above ground biomass (Mg ha^-1^) and woody productivity (Mg ha a^-1^) derived from 413 and 167 forest plots respectively, across lowland moist forest in South America.

Figure S8. Kriged maps of above ground biomass losses (Mg C ha^-1^ a^-1^) and stem mortality rates derived from 167 forest plots across lowland moist forest in South America.

Figure S9. Relationships between simulated mean *W_P_* and AGB from the 4 DGVMs, and precipitation and mean water deficit.

Figure S10. Relationships between simulated mean *W_P_* and AGB from the 4 DGVMs, and short wave radiation and temperature.
